# The orphan nuclear receptor NR4A2 is part of a p53–microRNA-34 network

**DOI:** 10.1038/srep25108

**Published:** 2016-04-28

**Authors:** Jordan A. Beard, Alexa Tenga, Justin Hills, Jessica D. Hoyer, Milu T. Cherian, Yong-Dong Wang, Taosheng Chen

**Affiliations:** 1 Department of Chemical Biology & Therapeutics, St. Jude Children’s Research Hospital, Memphis, Tennessee, USA; 2 Integrated Biomedical Sciences Program, University of Tennessee Health Science Center, Memphis, Tennessee, USA; 3 Department of Computational Biology, St. Jude Children’s Research Hospital, Memphis, Tennessee, USA

## Abstract

Nuclear receptor subfamily 4 group A member 2 (*NR4A2*) is an orphan nuclear receptor that is over-expressed in cancer and promotes cell proliferation, migration, transformation, and chemoresistance. Increased expression and function of NR4A2 have been attributed to various signaling pathways, but little is known about microRNA (miRNA) regulation of *NR4A2* in cancer. To investigate the posttranscriptional regulation of *NR4A2*, we used a 3′ untranslated region (UTR) reporter screen and identified miR-34 as a putative regulator of *NR4A2*. By using computer predictions, we identified and confirmed an miRNA recognition element in the 3′ UTR of *NR4A2* that was responsible for miR-34–mediated suppression. We next demonstrated that overexpression of exogenous miR-34 or activation of the p53 pathway, which regulates endogenous miR-34 expression, decreased NR4A2 expression. Consistent with previous reports, overexpression of *NR4A2* blocked the induction of p53 target genes, including *mir-34a*. This was a phenotypic effect, as *NR4A2* overexpression could rescue cells from p53-induced inhibition of proliferation. In summary, our results are the first characterization of a cancer-related miRNA capable of regulating *NR4A2* and suggest a network and possible feedback mechanism involving p53, miR-34, and NR4A2.

The nuclear receptor (NR) superfamily is a group of ligand-regulated transcription factors that control specific gene activity. NRs are thus important drug targets^1^,^2^. The 48 members of the human NR family share a common modular structure that includes an N-terminal domain containing an activation function 1 (AF-1) region, a DNA-binding domain (DBD), a hinge region, and a C-terminal ligand-binding domain (LBD) that also harbors an AF-2 domain. NR subfamily 4 group A (NR4A) consists of three highly homologous NRs, including *NR4A2* (also called Nurr1, NOT, TINUR, or NGFI-Bβ), that are characterized as immediate-early genes induced by mitogens, growth factors, and other stimuli^3^. The NR4A receptors have been implicated as having roles in multiple tissues and diseases, including cancer^4^, and their expression and function are associated with various oncogene and tumor suppressor pathways^5^.

NR4A2 is involved in cancer progression through a mechanism that has yet to be fully described. Most research indicates that NR4A2 has an oncogenic-like role, as it mediates cell proliferation, survival, transformation, invasion, and migration^6^,^7^,^8^,^9^,^10^. NR4A2 is highly expressed in squamous cell carcinoma (SCC), as compared to normal patient tissues, and prostaglandin-mediated induction of NR4A2 expression in SCC leads to increased resistance to 5-fluorouracil (5-FU)^11^. The effects of NR4A2 on chemoresistance are also seen in colorectal cancers^12^,^13^, and high expression of NR4A2 predicts a poor outcome for gastric cancer patients receiving 5-FU therapy^14^. NR4A2 may mediate these effects through a previously described NR4A2-p53 interaction that serves to suppress p53 transactivation, thereby protecting cells from p53-induced apoptosis^15^. Another NR4A family member, NR4A1 (also called Nur77, TR3, or NGFI-Bα), has also been implicated in p53 suppression^16^.

As the main arbiter of cell cycle progression, DNA repair, and apoptosis, p53 is a central hub for regulating tumor suppression. These effects are mediated through p53 target genes, which include the microRNA-34 (miR-34) family^17^,^18^,^19^,^20^. MicroRNAs (miRNAs) are small, endogenous, noncoding RNAs that help regulate target gene networks by binding complementarily to the 3′ untranslated regions (UTRs) of target genes to degrade or prevent their translation into proteins^21^. The miR-34 family, which consists of three isoforms (miR-34a, miR-34b, and miR-34c) encoded by two p53 direct transcriptional target genes (*mir-34a* and *mir-34b/c*), is considered to be partly responsible for carrying out p53’s tumor suppressive function by targeting the 3′ UTRs of genes that are critical to the cell cycle and survival, such as *BCL2*, cyclins, cyclin-dependent kinases (CDKs), and *MYCN*^19^,^22^. In mice, *mir-34a* is expressed at higher levels than *mir-34b/c*, except in lung tissues, where *mir-34b/c* is dominantly expressed^19^. Although miR-34 is dispensable for p53 tumor suppression^23^, it is critical for enhancing p53 stability and activity through miR-34–mediated suppression of negative regulators of p53, such as Sirt1^24^ and Hdm4^25^. The miR-34 isoforms have been clinically characterized as tumor suppressors in multiple cancer types, often independent of p53 mutation, as in neuroblastoma, where *mir-34a* is commonly deleted^26^,^27^, or in other cancers characterized by epigenetic silencing of miR-34^28^,^29^.

Recently, miR-132 was reported to target mouse *Nr4a2*^30^,^31^; the first characterization of an miRNA targeting an NR4A member. In this study, to determine whether human *NR4A2* is regulated by miRNAs and investigate its oncogenic-like role, we used an miRNA screening approach to identify cancer-relevant posttranscriptional regulatory networks of *NR4A2*, a subject that has not been fully explored. We identified miR-34 as a direct regulator of *NR4A2* through a specific sequence in its 3′ UTR. Furthermore, we determined that elevated miR-34 levels, resulting from exogenous overexpression or endogenous induction in a p53-dependent manner, decreased the levels of NR4A2. Corroborating the reported NR4A2-mediated inhibition of p53^15^, we found that overexpression of NR4A2 inhibited the ability of p53 to activate its target genes, including *mir-34a*. Lastly, overexpression of NR4A2 attenuated the sensitivity of cells to the p53 activator Nutlin-3a. These data are consistent with previous findings and, for the first time, identify miR-34 as a direct negative regulator of *NR4A2*. Together, they reveal a novel regulatory network linking p53, miR-34, and NR4A2, in which p53 can overcome its inhibition by endogenous NR4A2 through upregulating miR-34.

## Results

### Identification of miRNAs directly targeting the 3′ UTR of *NR4A2*

**Figure 1:**
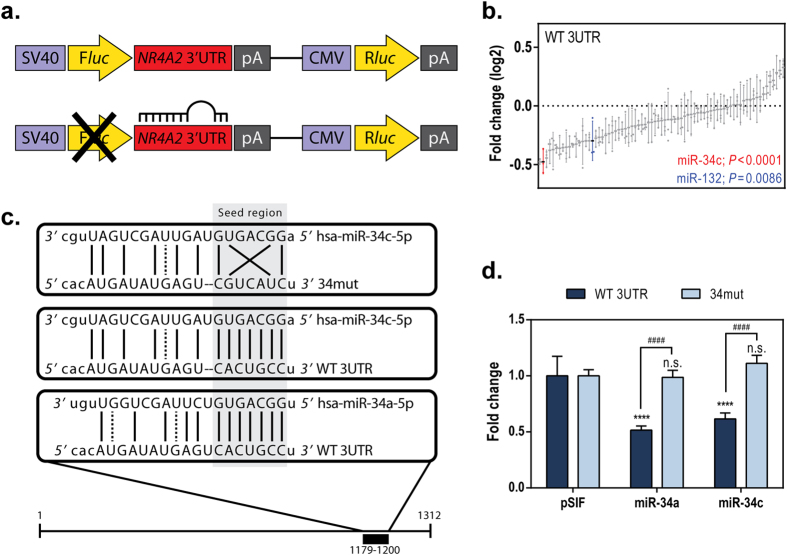
Screening for miRNAs that directly target the 3′ UTR of *NR4A2*. (**a**) A reporter plasmid containing the 3′ UTR of *NR4A2* downstream from a firefly luciferase (F*luc*) gene was used to identify miRNAs that putatively regulate *NR4A2*, relative to an internal *Renilla* luciferase (R*luc*) control gene. (**b**) 293T cells were transfected with the *NR4A2* 3′ UTR (WT 3UTR) reporter construct for 24 h. Transfected cells were reseeded in 96-well plates and reverse transfected with 75 individual cancer-relevant miRNAs. After 48 h of transfection, a Dual-Glo luciferase assay was performed, the ratio of F*luc*/R*luc* was calculated, and the log2 fold change was determined for each miRNA (*n* = 3), relative to a transfection control (pSIF, *n* = 9), and presented as a waterfall plot. The F*luc*/R*luc* value for pSIF was set as 1. Statistical significance was calculated using a one-way ANOVA and Dunnett’s test for multiple comparisons between pSIF and the indicated miRNAs. (**c**) A schematic of the predicted miR-34 seed region in the *NR4A2* 3′ UTR (WT 3UTR). This predicted miR-34 binding site in the *NR4A2* 3′ UTR was mutated, and the resulting loss of complementarity is shown (34mut). (**d**) WT 3UTR or 34mut reporter constructs were cotransfected with pSIF or the indicated miR-34 isoforms into HCT116 colorectal carcinoma cells for 72 h, after which a Dual-Glo luciferase assay was performed. The fold change of the F*luc*/R*luc* ratio with respect to pSIF was calculated (the F*luc*/R*luc* value of pSIF for each reporter transfection group was set as 1), and the statistical significance of the relation between pSIF and miR-34a or miR-34c was determined using a two-way ANOVA and Tukey’s test for multiple comparisons (****P ≤ 0.0001; n.s.*P* > 0.05). Statistically significant changes in WT 3UTR and 34mut for each miRNA transfection are indicated by ^####^(*P* ≤ 0.0001).

Increased *NR4A2* expression in cancer has been characterized as resulting from cell signaling events^5^, but miRNA-mediated regulation of *NR4A2* in cancer has hitherto been unexplored. To identify putative miRNAs capable of regulating *NR4A2* through its 3′ UTR, we assessed the effects of 75 cancer-relevant miRNAs by using a luciferase reporter system (Fig. 1a). Precursor miRNAs selected from an established library^32^ (see Supplementary Table S1) or an miRNA negative control (pSIF) were cotransfected with the *NR4A2* 3′ UTR reporter plasmid (WT 3UTR) in 293T cells. We observed that several miRNAs, including miR-34c, were capable of decreasing the luminescence reporter signal to a significantly greater extent than was miR-132, an miRNA that targets *Nr4a2*^30^,^31^ (log2 fold changes of −0.47521 and −0.29685, respectively; *P* ≤ 0.0001 and *P = *0.0086, respectively) (Fig. 1b and Table 1). This suggested that miR-34c was a modulator of *NR4A2* through its 3′ UTR.

By using three different miRNA prediction algorithm tools (TargetScan^33^, miRanda^34^, and PicTar^35^), we identified *NR4A2* as a predicted target of the miR-34 family (Table 1), further suggesting that this family of miRNAs were regulators of *NR4A2*. As expected, miR-132 was also predicted to target *NR4A2*. To corroborate the findings from “first-generation” prediction tools, we used the CoMeTa interactive database^36^, which integrates thousands of publicly available gene-expression datasets with the assumption that the predicted targets of an miRNA will be coexpressed with each other. The authors noted that targets falling within the top 50^th^ percentile of the CoMeTa analysis “co-rank” list for putative miRNA targets are highly predictive, based on previous dataset validation. CoMeTa analysis indicates that *NR4A2* falls within the top 40.568 and 39.182 percentiles for miR-34a and miR-34c-5p, respectively. By using the prediction algorithms, we identified a predicted miRNA recognition element complementary to the miR-34 seed region in the 3′ UTR of *NR4A2* and mutated this sequence to disrupt the complementarity in order to determine if there was direct regulation by miR-34 at this specific site (Fig. 1c). The wild-type 3′ UTR (WT 3UTR) reporter signal was effectively suppressed by cotransfection of miR-34a or miR-34c (52% and 62% of the control level, respectively; *P* ≤ 0.0001 for all) (Fig. 1d). The effect of miR-34a or miR-34c, compared to that of the control, was not significant when the miRNA was cotransfected with the 3′ UTR containing the seed-region mutation (34mut). Mutation of this predicted seed region was able to rescue the attenuation of the luminescence signal by miR-34a or miR-34c (Fig. 1d), increasing the signal from 52% to 99% and from 62% to 111% of the control level, respectively (*P* ≤ 0.0001 for all). These data indicate that miR-34 can target *NR4A2* at a specific seed complementarity region within its 3′ UTR. Furthermore, by analyzing a dataset from 97 patient samples with rectum adenocarcinoma, we found that the expression of *NR4A2* and miR-34a was inversely correlated to a similar extent as other miR-34 targets *AXL*^37^ and *SIRT1*^24^ (see Supplementary Figs S1 and S2). The read counts for miR-34b and miR-34c in this dataset were very low (data not shown), which is consistent with the known expression patterns of *mir-34b/c* primarily in lung tissues^19^, making correlation analyses for the *mir-34b/c* isoforms not suitable.

### miR-34 regulates endogenous NR4A2 levels

**Figure 2:**
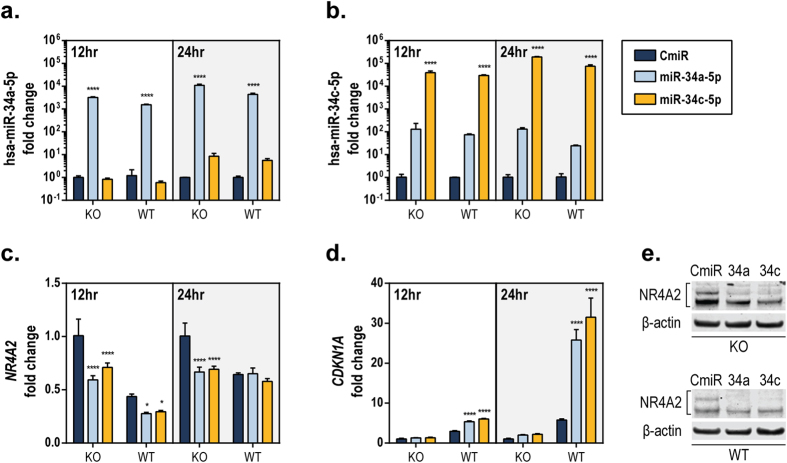
miRNA-34 downregulates endogenous NR4A2. Mature miR-34a and miR-34c mimics (10 nM) were transfected into HCT116^*TP53*−/−^ (KO) or HCT116 wild-type (WT) cells for 12 or 24 h. Expression of hsa-miR-34a–5p (**a**), hsa-miR-34c–5p (**b**), *NR4A2* (**c**), and *CDKN1A* (**d**) was determined using TaqMan qPCR probes. Mature miRNA expression was normalized to *RNU6B*, and *NR4A2* and *CDKN1A* were normalized to *GAPDH*. The value for the CmiR-transfected KO cells at each time point was set as 1. The statistical significance of the results of miR-34 transfections, compared to those of the control, in each cell line for each time point was calculated using a two-way ANOVA and Dunnett’s test for multiple comparisons. ****P ≤ 0.0001; *P ≤ 0.05. (**e**) Whole-cell lysates (45 μg) from HCT116^*TP53–/–*^ (KO) and HCT116 wild-type (WT) cells transfected for 48 h with control (CmiR), miR-34a (34a), or miR-34c (34c) mimics (10 nM) were assessed for expression of NR4A2 and β-actin protein by performing Western blotting analysis.

We next determined the *in vitro* effect of miR-34 on endogenous NR4A2 mRNA and protein levels through the use of miRNA mimics. Mature miR-34a-5p or miR-34c-5p mimics or a control (CmiR) were overexpressed in HCT116^*TP53*−/−^ (KO) or HCT116 wild-type (WT) isogenic cell lines to assess the direct effects of forced miRNA expression on *NR4A2*. Transfection of mature miR-34 mimics increased the expression of each miRNA (Fig. 2a,b). Transfection of miR-34a-5p slightly increased miR-34c-5p expression, probably because of specificity differences in the stem loop primers caused by the approximately 80% sequence homology of the two miR-34 isoforms. Overexpression of the miR-34a-5p mimic reduced the endogenous *NR4A2* expression to 59% and 64% of the control level in KO and WT cells, respectively, by 12 h post-transfection, whereas overexpression of the miR-34c-5p mimic reduced the *NR4A2* expression to 71% and 66% of the control level in KO and WT cells, respectively (Fig. 2c), and this effect was independent of p53 status. At 24 h post-transfection, the effect of the miR-34 mimics on *NR4A2* levels in HCT116 with wild-type p53 was no longer obvious, by comparison to the control. Additionally, the transfection of miR-34 mimics led to increased expression of the p53 target gene *CDKN1A*/p21 (Fig. 2d), increased p53 protein expression and acetylation, and subsequent p21 protein levels (see Supplementary Fig. S3) in wild-type HCT116 cells. The observation that increased levels of miR-34 led to enhanced p53 protein expression and activity probably reflects the known suppressive function of miR-34 on negative regulators of p53 protein stability (summarized in Supplementary Fig. S3)^24^,^25^, though *TP53* gene expression (in terms of mRNA levels) was also slightly increased by the miR-34 mimics (see Supplementary Fig. S3). The slight but significant increase in *TP53* expression induced by the miR-34 mimics is consistent with the observation that the levels of *NR4A2* correlate inversely with that of *TP53* gene expression, as discussed later. After 48 h of miR-34 mimic overexpression in HCT116 cells, the protein levels of NR4A2 (Fig. 2e) and another miR-34 target, Sirt1 (see Supplementary Fig. S3), were reduced in both KO and WT cells. Additionally, the effect of mature miR-34 mimics on *NR4A2* expression was assessed in another colorectal cancer cell line, RKO, with similar results observed on *CDKN1A* and *NR4A2* expression (see Supplementary Fig. S4). These data demonstrate that exogenous overexpression of a mature miR-34 decreases the levels of endogenous NR4A2 at both mRNA and protein level, regardless of the p53 status. The differential effect of exogenous miR-34 mimic on endogenous *NR4A2* may be attributable to the different levels of endogenous *NR4A2* mRNA in *TP53*+/+ and *TP53*−/− cells, although NR4A2 protein expression was substantially decreased in both *TP53*-isogenic backgrounds in response to exogenous miR-34 mimic. Additionally, we found that the protein level of NR4A2 was enhanced in RKO cells by a miR-34a inhibitor (see Supplementary Fig. S4), further supporting the regulation of NR4A2 by miR-34.

### p53 activation suppresses endogenous NR4A2 levels

**Figure 3:**
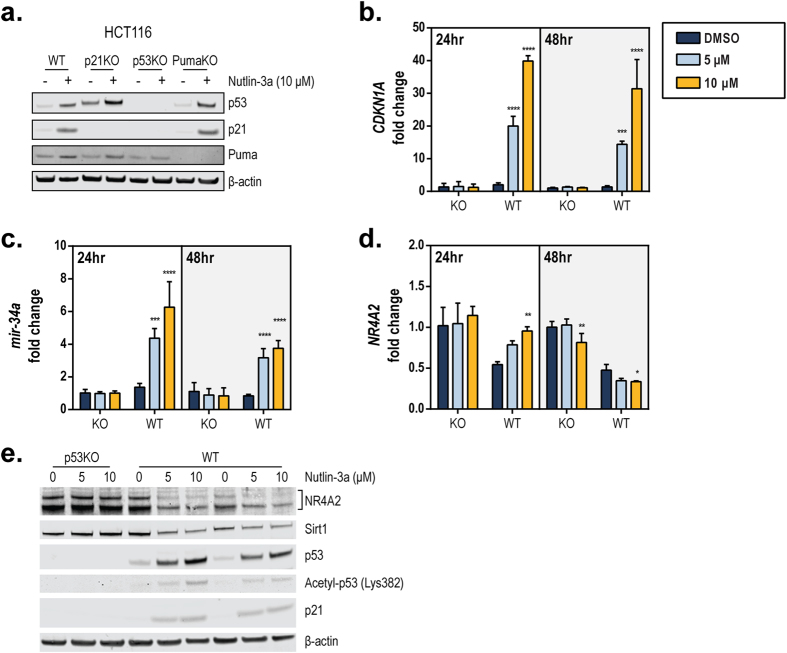
Nutlin-3a activation of p53 decreases endogenous NR4A2. (**a**) HCT116 wild-type (WT), HCT116^*CDKN1A−/−*^ (p21KO), HCT116^*TP53−/−*^ (p53KO), and HCT116^*BBC3−/−*^ (PumaKO) isogenic cell lines were treated for 48 h with 10 μM of Nutlin-3a or vehicle control (DMSO). Whole-cell lysates (40 μg) were assessed for expression of p53, p21, Puma, and β-actin by SDS-PAGE gel electrophoresis. (**b–d**) HCT116^*TP53−/−*^ (KO) and HCT116 wild-type (WT) cells were treated with vehicle control (DMSO) or Nutlin-3a (5 or 10 μM) for 24 or 48 h. Expression of *CDKN1A* (**b**), *mir-34a* (**c**), and *NR4A2* (**d**) was determined using TaqMan qPCR probes (normalized to *GAPDH*). The value for DMSO-treated KO cells at each time point was set as 1. The statistical significance of the results obtained with Nutlin-3a treatments, compared to those obtained with DMSO, in each cell line for each time point was calculated using a two-way ANOVA and Dunnett’s test for multiple comparisons. ****P ≤ 0.0001; ***P ≤ 0.001; **P ≤ 0.01; *P ≤ 0.05. (**e**) Whole-cell lysates (45 μg) from HCT116^*TP53*−/−^ (p53KO) and HCT116 wild-type (WT) cells treated for 48 h with DMSO (0 μM) or Nutlin-3a (5 or 10 μM) were assessed for expression of indicated proteins by performing Western blotting analysis.

As *mir-34a* is a direct transcriptional target of p53^17^,^20^, we next sought to determine if *NR4A2* expression was regulated by endogenous miR-34a in a p53-dependent manner. We treated cells with a chemical activator of p53, Nutlin-3a^38^, and confirmed that the p53 protein level increased after the treatment in cells expressing wild-type p53 (WT) but not in p53-deficient (KO) cells (Fig. 3a). As expected, the expression of transcriptional targets of p53, *CDKN1A*/p21 and *BBC3*/Puma, was also induced in a p53-dependent manner (Fig. 3a). HCT116^*CDKN1A*−/−^ and HCT116^*BBC3*−/−^ cells were used to demonstrate that Nutlin-3a acts upstream of *CDKN1A*/p21 and *BBC3*/Puma through p53 (Fig. 3a). We also found increased expression of *CDKN1A*/p21 and *mir-34a* in cells expressing wild-type p53 (Fig. 3b,c), accompanied by decreased *NR4A2* transcript levels after 48 h of treatment with Nutlin-3a (Fig. 3d). The NR4A family, including *NR4A2*, are immediate-early responsive genes that are inducible by many external stimuli^3^, and at an earlier time point (24 h), the levels of *NR4A2* mRNA were actually increased by Nutlin-3a (Fig. 3d). In agreement with mRNA expression, Nutlin-3a treatment for 48 h also caused a decrease in the NR4A2 protein expression in WT cells, but not in KO cells (Fig. 3e), further demonstrating the p53 requirement for Nutlin-3a–induced NR4A2 decrease. In addition to NR4A2, Nutlin-3a was also capable of decreasing the protein expression of Sirt1 (Fig. 3e), a known target of miR-34^24^, suggesting that p53 activation can regulate other downstream factors, possibly through miR-34. We confirmed these results in other colorectal cell lines (RKO and SW48) that are isogenic for *TP53* (see Supplementary Figs S5 and S6). These data indicate that p53 activation can induce the expression of *mir-34a*, while also decreasing the endogenous levels of NR4A2 and another known target of miR-34 (Sirt1). These results are consistent with the observation that the levels of miR-34 and NR4A2 are inversely correlated.

### Overexpression of *NR4A2* suppresses p53 activation

**Figure 4:**
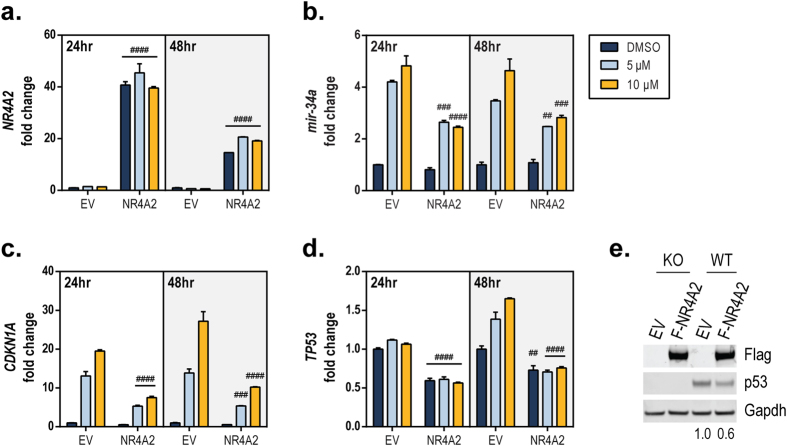
*NR4A2* overexpression suppresses p53 activation. HCT116 cells were transduced for 16 h with lentivirus expressing empty vector (EV) or 3xFlag-NR4A2 (NR4A2). The cell medium was then changed and the cells remained in culture for a total of 48 h. The cells were then reseeded and treated with vehicle (DMSO) or Nutlin-3a (5 or 10 μM) for 24 or 48 h. Expression of *NR4A2* (**a**) *mir-34a* (**b**) *CDKN1A* (**c**) and *TP53* (**d**) was determined using TaqMan qPCR probes (normalized to *GAPDH*). The value for DMSO-treated EV at each time point was set as 1. The statistical significance of the difference between the results with EV and NR4A2 for each treatment was determined for each time point by using a two-way ANOVA with Sidak’s multiple comparisons test. ^####^*P* ≤ 0.0001; ^###^*P* ≤ 0.001; ^##^*P* ≤ 0.01; ^#^*P* ≤ 0.05. (**e**) Whole-cell lysates from HCT116^*TP53*−/−^ (KO) and HCT116 wild-type (WT) cells transduced with lentivirus expressing EV or 3xFlag-NR4A2 (F-NR4A2) were assessed for expression of Flag (indicating NR4A2), p53, and Gapdh protein by performing SDS-PAGE gel electrophoresis. Relative p53 expression was determined using Odyssey Image Studio to calculate the ratio of p53 to Gapdh protein band density (displayed at the bottom of the gel). The ratio for EV in WT cells was set as 1.0.

It was previously reported that NR4A2 suppressed the transcriptional activity of p53 in the presence or absence of doxorubicin^15^. We sought to determine the effects of *NR4A2* overexpression on the induction of p53 activation by Nutlin-3a. HCT116 cells were transduced with lentivirus expressing empty vector control (EV) or 3xFlag-NR4A2, and overexpression of *NR4A2* was confirmed (Fig. 4a). The EV- or 3xFlag-NR4A2–transduced cells were treated with Nutlin-3a to determine the effect on p53 downstream target genes. Upon Nutlin-3a treatment, overexpression of *NR4A2* led to a significant attenuation of the p53-induced expression of the target genes *mir-34a* and *CDKN1A*/p21 (Fig. 4b,c). To assess the effect of NR4A2 on the binding of p53 to target gene promoters, we performed a chromatin immunoprecipitation (ChIP) assay. As shown in Supplementary Fig. S7, NR4A2 did not decrease p53 occupancy at the target gene promoters^39^,^40^, suggesting that another mechanism is responsible for the inhibitory effect of NR4A2 on p53. However, HCT116 cells overexpressing NR4A2 had diminished levels of p53 mRNA and protein (Fig. 4d,e and Supplementary Fig. S7). These data demonstrate that NR4A2 exerts inhibitory effects on the levels of p53 and its transcriptional target genes, which is consistent with previously reported results^15^ and suggests that the attenuation of p53 mRNA and protein levels, but not of p53 binding to target gene promoters, probably contributes to such inhibitory effects.

### Knockdown of NR4A2 enhances p53 activation

**Figure 5:**
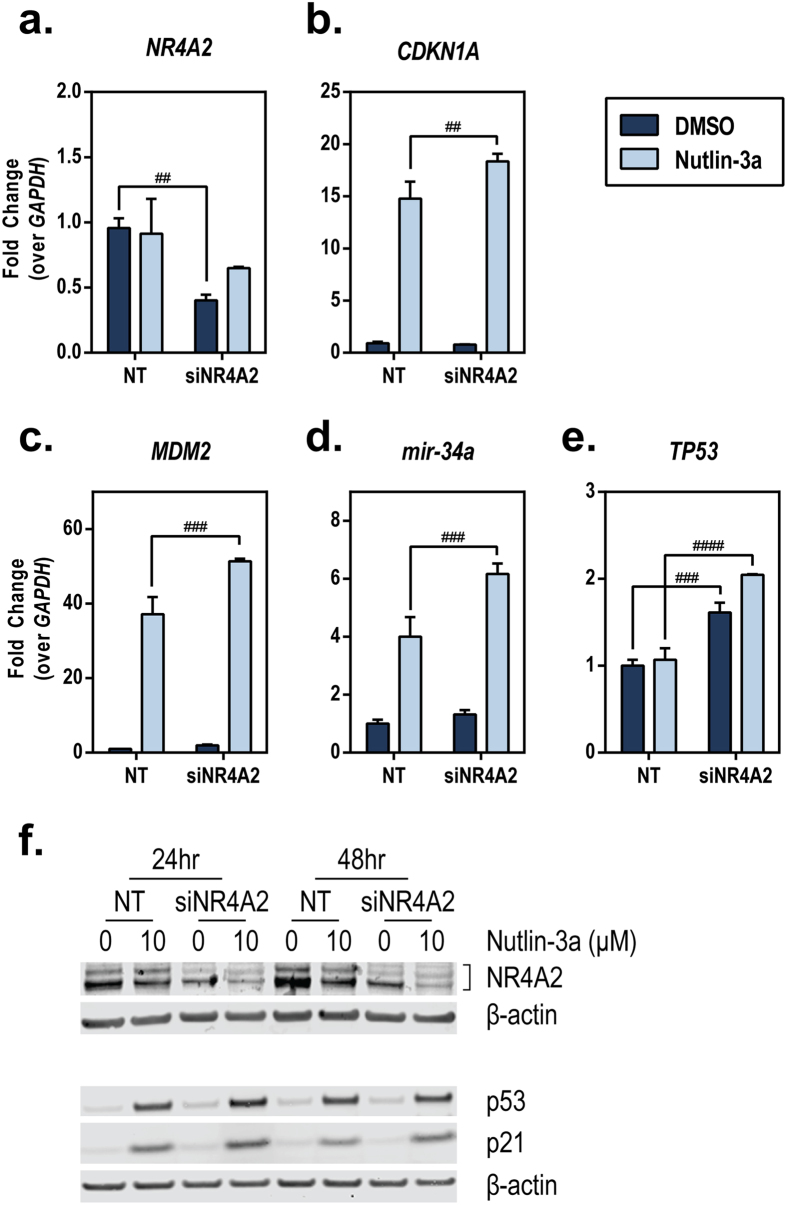
Knockdown of NR4A2 enhances p53 activation. HCT116 cells were transfected for 24 h with nontargeting control (NT) or siRNA targeting *NR4A2* (siNR4A2). The culture medium was then changed, and the cells remained in culture for a total of 48 h. The cells were then treated with vehicle (DMSO) or Nutlin-3a (10 μM) for 24 h. Expression of *NR4A2* (**a**) *CDKN1A* (**b**) *MDM2* (**c**) *mir-34a* (**d**) and *TP53* (**e**) was determined using TaqMan qPCR probes (normalized to *GAPDH*). The value for DMSO-treated NT was set as 1. The statistical significance of the results was determined using a two-way ANOVA with Tukey’s multiple comparison test. The significance of the differences within each transfection group between transfection groups (^####^*P* ≤ 0.0001; ^###^*P* ≤ 0.001; ^##^*P* ≤ 0.01) is represented. (**f**) HCT116 cells were transfected with NT or siNR4A2 as indicated above, followed by treatment of DMSO or Nutlin-3a (10 μM) for indicated length of times. Whole-cell lysates (45 μg) were assessed for expression of indicated proteins by performing Western blotting analysis.

To further confirm the inhibitory effect of NR4A2 on p53 transcriptional activity, we investigated the effects of reduced endogenous *NR4A2* levels on Nutlin-3a–induced p53 transcriptional target gene expression. HCT116 cells were transfected with nontargeting control (NT) or siRNA targeting *NR4A2* (siNR4A2), and knockdown of *NR4A2* was confirmed (Fig. 5a). After 48 h of siRNA-mediated knockdown, the cells were treated with Nutlin-3a for an additional 24 h to determine the effects of *NR4A2* knockdown on p53 target gene expression. We found that knocking down *NR4A2* caused an enhancement of Nutlin-3a–induced expression of *CDKN1A*/p21, *MDM2*, and *mir-34a* (Fig. 5b–d). Consistent with the inhibitory effect of overexpressed NR4A2 on p53 gene expression (Fig. 4d), knocking down *NR4A2* led to an increase in *TP53*/p53 gene expression for both the DMSO- and Nutlin-3a–treated groups (Fig. 5e). We also confirmed the reduced NR4A2 protein levels by the presence of siNR4A2 (Fig. 5f). Together, these data further suggest that NR4A2 can suppress p53 transcriptional activity, at least in part, by inhibiting the expression of p53 itself.

### Overexpression of *NR4A2* attenuates Nutlin-3a sensitivity

**Figure 6:**
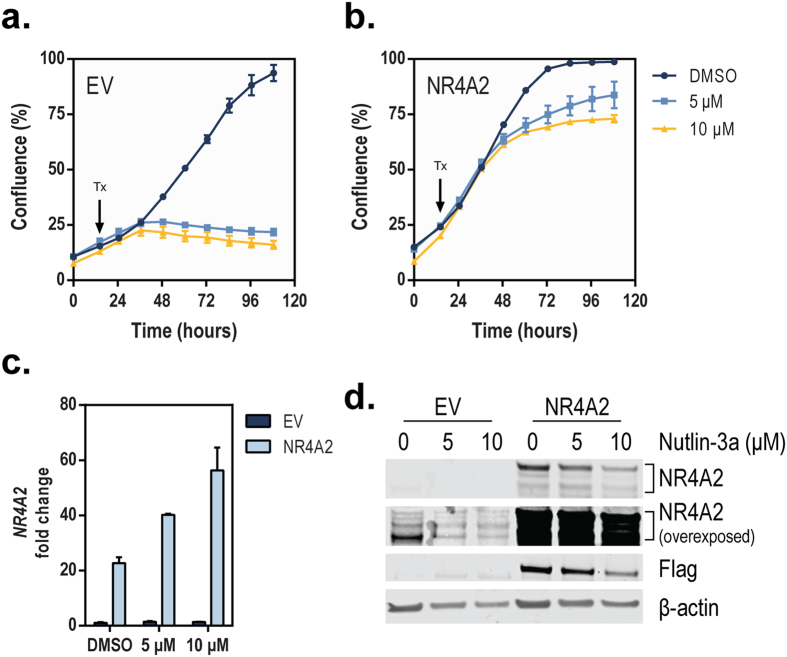
Overexpression of NR4A2 attenuates Nutlin-3a sensitivity. HCT116 cells were transduced for 16 h with lentivirus expressing empty vector (EV) or 3xFlag-NR4A2 (NR4A2). The cell medium was then changed, and the cells remained in culture for a total of 48 h. The cells were then reseeded into plates and the cell confluence was monitored using an IncuCyte ZOOM imaging system. After 14 h, the cell medium was replaced by medium containing vehicle (DMSO) or Nutlin-3a (5 or 10 μM) (indicated by Tx and arrow). The cell confluence for EV-expressing (**a**) or NR4A2-expressing (**b**) cells was monitored for a total of 108 h. (**c**) The cells were then collected and expression of *NR4A2* was determined using TaqMan qPCR probes (normalized to *ACTB*). The value for DMSO-treated EV was set as 1. (**d**) Total protein lysates (35 μg) were assessed for expression of NR4A2 (low and high exposure), Flag (to indicate overexpressed NR4A2), and β-actin.

We next examined the cellular effects of NR4A2-mediated suppression of p53 after Nutlin-3a treatment. HCT116 cells transduced for 48 h with lentivirus expressing empty vector control (EV) or 3xFlag-NR4A2 were plated in normal growth medium, and the cell proliferation was monitored using live-cell imaging. After 14 h, the cell culture medium was replaced with fresh medium containing vehicle (DMSO) or Nutlin-3a (5 or 10 μM), and the monitoring of cell proliferation continued. In EV-expressing cells, the inhibitory effect of Nutlin-3a on cell proliferation was substantial (Fig. 6a). In cells overexpressing *NR4A2*, Nutlin-3a–induced inhibition of cell proliferation was attenuated at both concentrations of Nutlin-3a tested (Fig. 6b). At the end of the real-time monitoring period (108 h), the cells were collected and their mRNA and protein levels were examined to confirm that NR4A2 expression was increased in cells transduced with *NR4A2*-expressing lentivirus (Fig. 6c,d). These data indicate that overexpression of NR4A2 attenuates the inhibitory effect of Nutlin-3a on cell proliferation. Together, our data are consistent with the known oncogenic role of NR4A2 and, for the first time, identify miR-34 as a negative regulator of *NR4A2* and reveal a novel functional network linking p53, miR-34, and p53.

## Discussion

Over the past decade, it has become increasingly evident that miRNA dysregulation plays an important role in human disease, including the development, progression, and therapeutic resistance of cancer. This process can be quite complex, as overlapping miRNA-mRNA networks can be formed, with a single miRNA having multiple targets or a single mRNA being a target for multiple miRNAs^41^. Research on miRNA regulation and the validation of biological targets has continued to increase in an effort to understand the multiple cellular pathways that are affected by specific miRNAs, and this area remains of particular interest as additional miRNA-based therapies are investigated and placed into clinical trials^42^,^43^.

**Figure 7:**
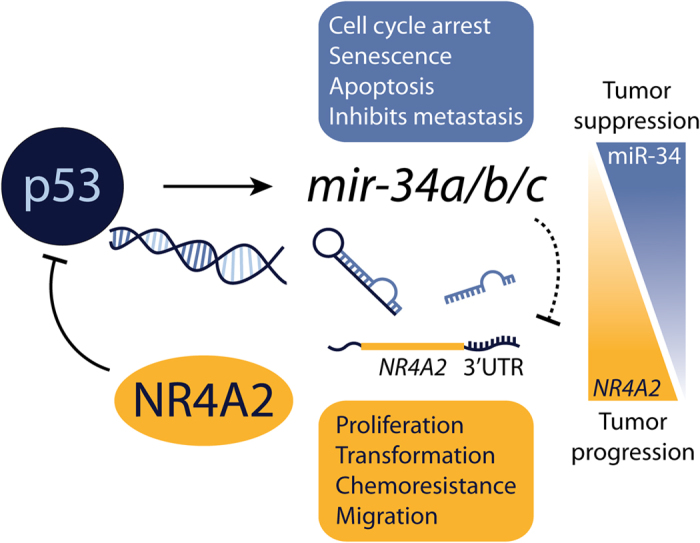
Graphical summary. Here, we describe a novel miR-34 regulatory mechanism of *NR4A2* that can act downstream of p53 activation. Additionally, our studies extend the finding that NR4A2 can suppress p53 expression and transcriptional targets, including Nutlin-3a–induced activation of *mir-34a*. The p53–miR-34 regulation of *NR4A2* may serve as a protective mechanism to prevent p53 suppression by NR4A2, in addition to suppressing other tumorigenic properties of NR4A2. The long arrow indicates activation; the blunt arrows indicate inhibition; the blunt arrow with the dotted line highlights the inhibition of *NR4A2* by miR-34 that indirectly activates p53. As tumors lose expression of mir-34, the expression and function of NR4A2 may become enhanced, shifting the balance from tumor suppression to tumor progression.

The NR4A family of orphan nuclear receptors has been studied extensively in various cancer models, and its regulation and function have been connected to multiple oncogenic and tumor suppressive pathways^5^. However, the contribution of miRNAs to the expression of the NR4A family is unclear. In this report, we have presented a novel p53–miR-34 regulatory mechanism of the orphan nuclear receptor NR4A2, in which miR-34 directly and negatively regulates NR4A2, which is itself able to repress p53-induced gene expression to rescue the inhibition of cell proliferation (Fig. 7). This is the first miRNA to be characterized as targeting the human NR4A2^30^,^31^.

Based on the known roles of NR4A2 in oncogenic processes within cancer, we posited that a likely candidate miRNA would be one that harbored tumor-suppressive functions and was downregulated in cancer, consequently leading to upregulation of NR4A2. As presented here, we identified and described the direct regulation of NR4A2 by miR-34, a well-described tumor suppressor–like miRNA that targets genes involved in cancer progression and is increasingly being exploited for its therapeutic advantage^22^. Whereas other putative miRNAs were identified as targeting *NR4A2*, we focused on the miR-34 family because of the known role of NR4A members in inhibiting p53^15^,^16^ and the positive regulation of miR-34 by p53^18^, thereby framing our discovery of the regulation of NR4A2 by miR-34 as a possible feedback mechanism. The miR-34 isoforms, predominantly *mir-34a*, have tumor suppressor functions in multiple cancer types, which is sometimes attributed to their p53 status^44^,^45^, though the mechanisms of chromosomal deletion or epigenetic silencing are also major contributors^26^,^27^,^28^,^29^, and miR-34 expression is prognostic for patient outcome or relapse^45^,^46^,^47^,^48^. Likewise, several studies have demonstrated the ability of miR-34a restoration to sensitize cells to chemotherapeutic agents, including erlotinib^49^, Adriamycin/doxorubicin^50^, and 5-FU^51^. By using a colorectal cancer cell line pair that was sensitive or resistant to 5-FU, Akao *et al*. demonstrated that miR-34a was significantly downregulated in the resistant cells, and this was accompanied by increased expression of *SIRT1*, a target of miR-34. The expression of endogenous miR-34a remained low in 5-FU–resistant cells treated with 5-FU, and the restoration of miR-34a or knockdown of *SIRT1* in the resistant cells overcame their resistant phenotype. In light of this observation, it would be interesting to study the relationship of miR-34 and NR4A2 with respect to chemoresistance, as increased NR4A2 expression is correlated with worse patient outcomes with regard to 5-FU therapy^11^,^12^,^13^,^14^.

By using an *in vitro* luminescence reporter-based screening assay and three computer-based prediction algorithms^33^,^34^,^35^,^36^, we identified miR-34c as a putative regulator of *NR4A2* via its 3′ UTR region (Fig. 1 and Table 1). This regulatory effect was confirmed by using mutagenesis of the predicted miRNA recognition element that is complementary to the miR-34 seed region. Other miRNAs from this screen have been evaluated using computer-based and mutagenesis approaches (Table 1 and unpublished data), and the functional significance of these miRNA regulators of *NR4A2* remains to be elucidated in future studies. Using publically available data, we found a weak negative (inverse) correlation of miR-34a with *NR4A2* (see Supplementary Figs S1 and S2), which was similar to the correlations of miR-34a with other known miR-34 target genes, such as *AXL*^37^ and *SIRT1*^24^, although *NOTCH1*^50^ did not show a negative correlation as expected. Interestingly, the patient sample data demonstrated two populations of *TP53*-expressing cells, and when we considered only those patient tumor samples with normal levels of *TP53*, the correlation became stronger. As we would hypothesize, there was a positive correlation of *NR4A2* with *AXL* and *SIRT1* since these genes share a common miRNA regulator, miR-34. Consistent with our data in Figures 4d and 5e, the level of *NR4A2* was negatively correlated to that of *TP53* (see Supplementary Fig. S2).

The next objective was to determine whether miR-34 could regulate endogenous NR4A2, at both mRNA and protein levels. Indeed, overexpression of mature miR-34 isoforms reduced the endogenous levels of NR4A2 mRNA and protein in both wild-type and p53-deficient cells (Fig. 2 and Supplementary Fig. S4). This would be expected, as forced expression of exogenous miR-34 bypasses the requirement for p53. Additionally, miR-34 overexpression enhanced the p53 activity in wild-type cells, probably because of the known regulatory effect of miR-34 on negative regulators of p53 (Fig. 2 and Supplementary Fig. S3). This effect is largely the result of increased p53 protein levels rather than substantial changes in *TP53* transcript levels (see Supplementary Fig. S3). The effect of miR-34 on *NR4A2* was not as dramatic in the HCT116 p53 wild-type cells, especially at a later time point (24 h) (Fig. 2c), possibly because the lower levels of *NR4A2* in these cells no longer respond to exogenous miR-34 expression after the initial transfection, although reduced protein expression was observed at 48 h post-transfection (Fig. 2e). This effect could also result from the delicate balance of *NR4A2* expression being regulated directly by miR-34 or indirectly through cellular stress. Correspondingly, RKO p53 wild-type cells had higher levels of endogenous *NR4A2* and responded better to miR-34 mimics than their p53-deficient counterpart (Supplementary Fig. S4), further supporting the notion that endogenous levels of *NR4A2* affect its response to exogenous miR-34.

To further understand the importance of miR-34 regulation of *NR4A2* in the cellular context, we examined the relationship between endogenous miR-34 and endogenous *NR4A2* by using our isogenic cell line pairs that possess wild-type or deleted *TP53*. One interesting observation was that treating HCT116 cells with Nutlin-3a at an early time point (24 h) led to increased *NR4A2* expression, but the opposite effect was observed at a later time point (48 h) (Fig. 3d). Consistent with this result, the NR4A2 protein expression was also reduced after 48 h of Nutlin-3a treatment in WT cells (Fig. 3e). The NR4A family are immediate-early genes that are inducible by many stimuli, including cell stress and cytotoxic agents^3^, whereas Nutlin-3a has a p53-independent role and can induce a DNA damage response leading to cell cycle arrest^52^. One of these properties might account for the earlier induction of *NR4A2* by Nutlin-3a, as the NR4A family mediates DNA double-strand break repair^53^. This hypothesis could be further tested by using *MDM2*-deficient cells to determine if these effects are independent of the primary role of Nutlin-3a in Mdm2 inhibition.

Finally, we investigated the previously reported inhibitory effect of NR4A2 on p53 activation^15^ to determine if this regulation had an effect in the cellular context. We confirmed that overexpression of exogenous *NR4A2* can inhibit p53 induction of target genes in response to Nutlin-3a treatment (Fig. 4) and that the opposite effect is seen when *NR4A2* expression is reduced by using siRNA (Fig. 5). Our data indicate that this effect might be achieved at least partially through NR4A2 suppression of *TP53*/p53 gene expression. Further investigation is warranted to determine if this suppression results from direct or indirect repression of the *TP53* promoter. The suppressive effect of NR4A2 on the expression of p53 and its transcriptional targets was not reflected in changes in the binding of p53 to its target gene promoters. However, this finding may reflect the sensitivity of the assay, as even in the presence of NR4A2, enough p53 may be present to saturate the promoter response elements during Nutlin-3a treatment. It is also possible that NR4A2 is not inhibitory to p53 binding but is repressive of p53 transactivation at its target gene promoters.

NR4A2 inhibition of the p53 response has previously been investigated only with respect to the genotoxic agent doxorubicin; here, we have demonstrated a similar effect using a more targeted p53 activator. When cells overexpressing *NR4A2* were subjected to prolonged treatment with Nutlin-3a, we observed a substantial rescue of cells from Nutlin-3a–induced inhibition of proliferation (Fig. 6), suggesting that the inhibitory effect of *NR4A2* on p53 activation is responsible for phenotypic observations that are indicative of p53-induced tumor suppression. Interestingly, in cells overexpressing *NR4A2, NR4A2* expression was increased in Nutlin-3a–treated cells after prolonged treatment (Fig. 6c). This effect may be attributable to the ability of *NR4A2* to repress the inhibition of cell proliferation, and as the selective pressure of Nutlin-3a is applied to all the cells, those cells expressing the highest levels of *NR4A2* persist in culture.

In summary, our study identified and confirmed a novel regulation of NR4A2 by miR-34. This regulatory effect is observed in the context of p53 activation, and NR4A2 itself is able to repress the p53 response. These events can be regarded as a positive feedback loop for p53 (Fig. 7), much like other miR-34 targets that also suppress p53^24^,^25^. In tumors expressing wild-type p53, this can serve as a means by which p53 can release itself from repression by NR4A2, further enhancing the p53 response. Alternatively, cells that contain p53 mutations or that overexpress *NR4A2* through other means may lack the p53-mediated suppression of NR4A2, allowing NR4A2 to perform other oncogenic activities through its role as a transcription factor. As tumors lose expression of *mir-34*, through promoter methylation or the loss of p53, the expression and function of NR4A2 may become enhanced, shifting the balance from tumor suppression to tumor progression (Fig. 7), though this may not be directly traceable because of the complexity of miRNA-mRNA regulatory networks.

## Materials and Methods

### Cell culture

All cell lines were grown in culture at 37 °C in 5% CO_2_. Human embryonic kidney 293 T cells (ATCC, Manassas, VA) were grown according to the manufacturer’s protocol. Human colorectal carcinoma HCT116 wild-type and *TP53*−/− isogenic cell lines, which were derived from an adult male harboring a mutation in codon 13 of the ras proto-oncogene, were obtained from the Genetic Resources Core Facility at Johns Hopkins University School of Medicine (Baltimore, MD) and grown in McCoy’s 5A medium (ATCC) supplemented with 10% fetal bovine serum (Thermo Fisher Scientific Inc., Rockford, IL) and 1% penicillin-streptomycin (Life Technologies, Carlsbad, CA). RKO human colon carcinoma (derived from a female harboring mutations in BRAF, NF1 and PIK3CA) and SW48 human colorectal adenocarcinoma (derived from a female harboring mutations in CTNNB1, FBXW7 and EGFR) (wild-type and *TP53*−/−) and HCT116 (*CDKN1A*−/− and *BBC3*−/−) isogenic cell line pairs^54^,^55^,^56^ were obtained from Horizon Discovery (Cambridge, UK) and grown according to the manufacturer’s protocol. Cells collected for RNA and protein extraction were detached with 0.05% trypsin-EDTA (Life Technologies). For all luminescence-based assays, the cells were plated using phenol-red–free DMEM or RPMI-1640 medium (Life Technologies).

### 3′ UTR reporter plasmid and microRNA screen

The *NR4A2* 3′ UTR was cloned by GeneCopoeia, Inc. (Rockville, MD), directly downstream from a firefly luciferase (F*luc*) gene under the control of an SV40 promoter in the pEZX-MT01 vector, which also contains a *Renilla* luciferase (R*luc*) gene under the control of a CMV promoter (as a transfection control). This reporter construct (WT 3UTR) was used to identify miRNAs that regulate F*luc* activity through binding to the *NR4A2* 3′ UTR and degradation or translational inhibition of fused F*luc* mRNA. The R*luc* activity was used to normalize the F*luc*. The 293T cells were cotransfected for 48 h with the 3′ UTR reporter plasmid and 75 cancer-related miRNAs selected from a previously described library^32^ (see Supplementary Table S1) by using Lipofectamine 2000 (Life Technologies) according to the manufacturer’s protocol. Fugene 6 (Promega, Madison, WI) was used for transient cotransfection of reporter gene plasmids and miRNAs into HCT116 cells according to the manufacturer’s instructions. Dual-Glo luciferase assays (Promega, Madison, WI) were performed to measure and calculate the ratios of firefly and *Renilla* luciferase activity. Luciferase activity was measured with an EnVision 2102 Multilabel Plate Reader (Perkin Elmer Inc., Waltham, MA).

### miRNA target prediction and mutagenesis

Three miRNA target-prediction algorithms were used to identify putative miRNA regulators of *NR4A2*: TargetScanHuman, http://www.targetscan.org33; miRanda, http://www.microrna.org34; and PicTar, http://pictar.mdc-berlin.de/35. By using these algorithms, a putative seed region was determined and mutated using site-directed mutagenesis (Mutagenex, Inc., Piscataway, NJ). Reporter constructs containing either the wild-type (WT 3UTR) or mutated (34mut) 3′ UTR were used to demonstrate miR-34 specificity in the *NR4A2* 3′ UTR.

### Molecular cloning

NR4A2 cDNA was cloned into the pEXM12-3xFLAG (N-terminal) vector (GeneCopoeia, Inc.). We used the primers listed in Supplementary Table S2 to amplify 3xFLAG-NR4A2 cDNA from pEX-3xFLAG-NR4A2 by PCR then subcloned it into a pSin-EF2-IRES-Blast lentiviral expression vector (kindly provided by Dr. Mark E. Hatley). All DNA constructs were confirmed by Sanger sequencing. Lentiviral expression plasmids were packaged into viral particles using the psPAX2 packaging (Addgene plasmid #12260) and pMD2.G envelope (Addgene plasmid #12259) vectors.

### miRNA mimics and inhibitor, RNAi, and chemical treatments

Exogenous expression or inhibition of mature miRNAs was performed with *mir*Vana mimics (CmiR: cat# 446058; miR-34a: cat# 446066; miR-34c: cat# 446066) or inhibitors (Cntrl. inhib.: cat# 446076; miR-34a inhib.: cat# 446084; used at 10 nM) transfected using Lipofectamine RNAiMAX reagent (Ambion, Life Technologies). All chemical treatments were performed in full growth medium containing 0.1% dimethyl sulfoxide (DMSO; as a negative control) (Thermo Fisher Scientific) or Nutlin-3a (Sigma Aldrich) as specified by the experimental design. Small interfering RNA (siRNA) was obtained from Dharmacon (Lafayette, CO) and transfected at a concentration of 20 nM using Lipofectamine RNAiMAX reagent.

### RNA extraction and quantitative real-time PCR

RNA was extracted using Maxwell simplyRNA kits and a Maxwell 16 Instrument (Promega). For the experiments that used miRNA mimics, the total RNA (including small RNAs) was extracted from the collected cells using miRNeasy Mini kits (Qiagen) in accordance with the manufacturer’s instructions. RNA concentrations were measured using a NanoDrop 8000 UV-Vis Spectrophotometer (Thermo Fisher Scientific). All cDNA used in mRNA and miRNA quantitative real-time PCR (qPCR) analyses was synthesized from extracted RNA by using the SuperScript VILO cDNA synthesis kit (Life Technologies) according to the manufacturer’s protocol. The mRNA and miRNA expression data were generated using Applied Biosystems TaqMan assays (20×) and Fast Advanced Master Mix (Life Technologies). Thermal cycling for qPCR was performed with an Applied Biosystems 7900HT Fast Real-Time PCR system (Life Technologies) in accordance with the TaqMan Fast protocol.

### Protein extraction and Western blot analysis

Protein was isolated by incubating cells in Pierce RIPA lysis buffer with added Halt Protease Inhibitor cocktail (Thermo Fisher Scientific) for 30 min on ice then sonicating the lysate for 10 s at 50% amplitude to shear the DNA. The protein concentration was measured using the Pierce BCA Protein Assay (Thermo Fisher Scientific) according to the manufacturer’s instructions.

Protein lysates were resolved on NuPAGE 4–12% SDS-PAGE gradient gels (Life Technologies). After electrophoresis was completed, the proteins were transferred from the gels to nitrocellulose membranes with an iBlot dry transfer system (Life Technologies). Protein gels used for detection of NR4A2 protein were transferred to polyvinylidene fluoride (PVDF) membranes using wet transfer for 1 h at 100 V constant voltage. The specific antibodies used were rabbit anti-NR4A2 (Santa Cruz Biotechnology, sc-5568; M-196; 1:500 dilution)^57^,^58^ (see Supplementary Fig. S8), mouse anti-p53 (Santa Cruz Biotechnology, sc-65334; B-P3), rabbit anti-Sirt1 (Santa Cruz Biotechnology, sc-15404; H-300), goat anti-PUMAα (Santa Cruz Biotechnology, sc-19187; N-19); rabbit anti–acetyl-p53 (Lys382) (Cell Signaling, Danvers, MA, #2525); mouse anti-Gapdh (Ambion, Life Technologies); mouse anti-p21 (Oncogene Research Products, Boston, MA, OP64); mouse anti–β-actin (Sigma Aldrich, A5441; clone AC-15); and mouse anti-FLAG (Sigma Aldrich, F1804; clone M2). All antibodies were diluted in Odyssey Blocking Buffer (LI-COR Biotechnology, Lincoln, NE). The secondary antibodies were goat anti-rabbit and goat anti-mouse IRDye 800WC or IRDye 680LT (LI-COR). All Western blot imaging was conducted using a LI-COR Odyssey Infrared Imaging System.

### Chromatin immunoprecipitation (ChIP)

HCT116 cells transduced with pSin-EF2-IRES-Blast empty vector (EV) or 3xFlag-NR4A2 lentivirus were grown in flasks and treated with vehicle (DMSO, 0.1%) or Nutlin-3a (10 μM) for 6 h. The chromatin was then crosslinked with 1% formaldehyde for 10 min. Cell extracts were digested for 10 min with 50 units of micrococcal nuclease (New England Biolabs, Ipswich, MA) at 37 °C and further sonicated to yield sheared DNA fragments with an average length of 200 to 1000 base pairs. The sonicated samples were centrifuged to pellet the cell debris, and the supernatant was diluted 7-fold with ChIP dilution buffer (0.01% SDS, 1.1% Triton X-100, 1.2 mM EDTA, 16.7 mM Tris-HCl, pH 8.1, 167 mM NaCl, and protease inhibitor cocktail). The samples were precleared with ChIP-grade Protein G agarose beads (Cell Signaling, #9007) in ChIP dilution buffer (1:1 ratio). Diluted supernatant (100 μL) was reserved as input (10%) for each treatment. Chromatin (1 mL) was used for each immunoprecipitation and was incubated overnight at 4 °C with mouse anti-p53 (Santa Cruz Biotechnology, sc-126; DO-1) or control mouse IgG. The antibody-protein-DNA complexes were precipitated by incubation with Protein G–agarose beads for 2 h at 4 °C. The protein-DNA complexes were eluted from the beads with elution buffer (1% SDS, 0.1 M NaHCO_3_). The crosslinks were reversed, and DNA was eluted from the protein-DNA complexes by adding 200 mM NaCl and incubating at least overnight at 65 °C. DNA was recovered and purified after protein digestion with Proteinase K at 45 °C for 2 h using the Qiagen DNeasy Blood & Tissue Kit (Qiagen Cat. No: 69506). Quantitative real-time PCR was performed to determine the changes in p53 occupancy at various known sites of p53 binding. The double-negative controls were nonspecific antibody (normal mouse IgG) and primers coding for regions that do not interact with p53. The thermal cycling conditions were 95 °C for 10 min followed by 45 cycles of 25 s at 95 °C, 30 s at 60 °C, and 30 s at 72 °C. The primers used are listed in Supplementary Table S2.

### Cell viability assays

Real-time cell growth in response to the various treatments was measured as the degree of cell confluence in culture plates and was determined using an IncuCyte ZOOM live-cell imaging system (Essen BioScience, Ann Arbor, MI). Cell proliferation curves were plotted using confluence values at specified time points for each treatment.

### Analyses of expression data from online databases

The RNASeq and miRNASeq dataset of 164 patients with rectum adenocarcinoma was acquired from the TGCA Research Network: http://cancergenome.nih.gov/. After log2 transformation of reported normalized_count for mRNA expression and read_per_million_miRNA_mapped for mature miRNA expression, a subset of 97 samples with data generated from the HiSeq platform (for a consistent and reliable comparison) was investigated. The correlations between the expression of miR-34a and *NR4A2*, as well as other published miR-34 targets—*AXL*^37^, *NOTCH1*^50^, and *SIRT1*^24^—and *TP53* were determined by the regression analysis using Stata software (College Station, TX).

### Statistical analysis

All experiments were performed at least three times, and the independent replicates are represented as mean ± standard deviation. Data normalization, statistical tests used, and representations of *P*-value are indicated for each figure in the corresponding legend.

## Additional Information

**How to cite this article**: Beard, J. A. *et al*. The orphan nuclear receptor NR4A2 is part of a p53–microRNA-34 network. *Sci. Rep.* **6**, 25108; doi: 10.1038/srep25108 (2016).

## Supplementary Material

Supplementary Information

## Figures and Tables

**Table 1:** miRNAs that putatively regulate *NR4A2* through its 3′ UTR.

Rank	miRNA ID	Log2 fold change	*P*-value	TargetScan	miRanda	PicTar	Other predicted *NR4A* genes	Other targets
1	hsa-miR-335	−0.47549 ± 0.0223	<0.0001	No	No	No	*NR4A3*	*BCL2L2, SOX4, RB1, RUNX2*
**2**	**hsa-miR-34c**	−0.4752 ± 0.1028	<0.0001	**Yes**	**Yes**	**Yes**	—	*BCL2, CCND1, CDK4/6, FRA1, MET, MYC, MYCN, SIRT1, SNAI1*
3	hsa-miR-144	−0.44443 ± 0.0696	<0.0001	No	No	No	*NR4A3*	*NOTCH1, PTEN, TGFB1*
**4**	**hsa-miR-214**	−0.37908 ± 0.0942	0.0002	No	**Yes**	**Yes**	*NR4A1*	*BCL2L2, EZH2, PTEN, TWIST1*
5	hsa-miR-191	−0.37123 ± 0.1471	0.0002	No	No	No	—	*CDK6, SOX4*
6	hsa-miR-15a	−0.36165 ± 0.0343	0.0004	No	No	No	*NR4A1, NR4A3*	*BCL2, CCND1, CCND2, CCNE1, CRKL, VEGFA*
7	hsa-miR-155	−0.36052 ± 0.0926	0.0004	No	No	No	*NR4A3*	*APC, FOXO3, MLH1, RUNX2, SMAD1, SMAD2, SMAD5*
**8**	**hsa-miR-20a**	−0.35512 ± 0.0296	0.0005	**Yes**	**Yes**	**Yes**	*NR4A3*	*CCND1, CDKN1A, E2F1, HIF1A, KIT, PTEN*
9	hsa-miR-25	−0.35227 ± 0.128	0.0006	No	No	No	*NR4A3*	*BCL2L11, CDH1, EZH2, MDM2, TP53*
**17**	**hsa-miR-132**	−0.296850 ± 0.1684	0.0086	**Yes**	**Yes**	**Yes**	—	*CDKN1A, NR4A2, RB1, SIRT1*

The log2 fold changes from the reporter assay screen of the top nine miRNAs and miR-132 are listed. The significance of each miRNA in comparison to the transfection control (pSIF) was determined using a one-way ANOVA and Dunnett’s test for multiple comparisons. Three online prediction algorithms (TargetScanHuman, www.targetscan.org; miRanda, www.microrna.org; and PicTar, pictar.mdc-berlin.de) were used to predict if a seed region match was present in the *NR4A2* 3′ UTR. The same algorithms were used to predict if any of the listed miRNAs targeted other NR4A members. The other known targets of each miRNA are listed (taken from miRTarBase, mirtarbase.mbc.nctu.edu.tw).
