# Supplementary material for: The orphan nuclear receptor NR4A2 is part of a p53–microRNA-34 network
Source: Sci Rep. 2016 Apr 28;6:25108. doi: 10.1038/srep25108 (PMC4848494; doi:10.1038/srep25108)
Supplement: Supplementary Information [file srep25108-s1.pdf]

Supplementary Information for

The orphan nuclear receptor NR4A2 is part of a p53−microRNA-34 network

Jordan A. Beard[1,2], Alexa Tenga[1,2], Justin Hills[1], Jessica D. Hoyer[1,2], Milu T. Cherian[1], Yong-Dong Wang[3], & Taosheng Chen[1,2]*

[1]Department of Chemical Biology & Therapeutics, St. Jude Children's Research Hospital, Memphis, Tennessee, USA.

[2]Integrated Biomedical Sciences Program, University of Tennessee Health Science Center, Memphis, Tennessee, USA.

[3]Department of Computational Biology, St. Jude Children's Research Hospital, Memphis, Tennessee, USA.

***Corresponding author:** Taosheng Chen, Department of Chemical Biology & Therapeutics, St. Jude Children's Research Hospital, 262 Danny Thomas Pl., MS 1000, Memphis, TN 38105, USA. Email: taosheng.chen@stjude.org; Tel.: +1-901-595-5937; Fax: +1-901-595-5715

Supplementary Table S1. List of miRNAs screened.

miRNA ID	mirBase v14.0 Accession #	Log2 fold change	Std. dev.	*P*-value	Significance
hsa-miR-335	MIMAT0000765	−0.475	0.0223	< 0.0001	****
hsa-miR-34c	MIMAT0000686	−0.475	0.1028	< 0.0001	****
hsa-miR-144	MIMAT0000436	−0.444	0.0696	< 0.0001	****
hsa-miR-214	MIMAT0000271	−0.379	0.0942	0.0002	***
hsa-miR-191	MIMAT0000440	−0.371	0.1471	0.0002	***
hsa-miR-15a	MIMAT0000068	−0.362	0.0343	0.0004	***
hsa-miR-155	MIMAT0000646	−0.361	0.0926	0.0004	***
hsa-miR-20a	MIMAT0000075	−0.355	0.0296	0.0005	***
hsa-miR-25	MIMAT0000081	−0.352	0.128	0.0006	***
hsa-miR-122	MIMAT0000421	−0.348	0.0902	0.0007	***
hsa-miR-140	MIMAT0000431	−0.333	0.015	0.0016	**
hsa-miR-17	MIMAT0000070	−0.325	0.0957	0.0024	**
hsa-let-7b	MIMAT0000063	−0.314	0.0205	0.0039	**
hsa-miR-363	MIMAT0000707	−0.303	0.1958	0.0066	**
hsa-miR-218	MIMAT0000275	−0.297	0.0494	0.0085	**
hsa-miR-9	MIMAT0000441	−0.297	0.0711	0.0086	**
hsa-miR-132	MIMAT0000426	−0.297	0.1684	0.0086	**
hsa-miR-21	MIMAT0000076	−0.274	0.0699	0.0224	*
hsa-miR-184	MIMAT0000454	−0.268	0.0717	0.0289	*
hsa-miR-32	MIMAT0000090	−0.266	0.0533	0.0317	*
hsa-miR-148a	MIMAT0000243	−0.251	0.1481	0.0553	ns
hsa-miR-10b	MIMAT0000254	−0.237	0.0745	0.0892	ns

hsa-miR-30c	MIMAT0000244	−0.228	0.138	0.1213	ns
hsa-miR-210	MIMAT0000267	−0.213	0.0814	0.1895	ns
hsa-miR-128b	MIMAT0031095	−0.203	0.0917	0.2513	ns
hsa-miR-92a	MIMAT0000092	−0.199	0.0536	0.2774	ns
hsa-miR-133b	MIMAT0000770	−0.195	0.1246	0.3096	ns
hsa-miR-143	MIMAT0000435	−0.184	0.1607	0.3967	ns
hsa-miR-150	MIMAT0000451	−0.173	0.1322	0.5083	ns
hsa-miR-29a	MIMAT0000086	−0.171	0.1333	0.5269	ns
hsa-miR-222	MIMAT0000279	−0.164	0.0358	0.5981	ns
hsa-miR-98	MIMAT0000096	−0.161	0.0807	0.6303	ns
hsa-miR-18a	MIMAT0000072	−0.158	0.046	0.6677	ns
hsa-miR-181b	MIMAT0000257	−0.143	0.1663	0.8227	ns
hsa-miR-27a	MIMAT0000084	−0.139	0.1611	0.8586	ns
hsa-miR-301a	MIMAT0000688	−0.132	0.1036	0.9086	ns
hsa-miR-130a	MIMAT0000425	−0.127	0.0528	0.9394	ns
hsa-miR-206	MIMAT0000462	−0.126	0.1139	0.9474	ns
hsa-let-7e	MIMAT0000066	−0.119	0.1312	0.9705	ns
hsa-miR-127	MIMAT0004604	−0.117	0.2329	0.9794	ns
hsa-miR-205	MIMAT0000266	−0.114	0.113	0.9807	ns
hsa-miR-23b	MIMAT0000418	−0.113	0.0235	0.9812	ns
hsa-miR-19a	MIMAT0000073	−0.112	0.1182	0.982	ns
hsa-miR-212	MIMAT0000269	−0.106	0.1397	0.9843	ns

hsa-miR-34a	MIMAT0000255	−0.089	0.2004	0.9983	ns
hsa-miR-126	MIMAT0000445	−0.078	0.1309	0.9986	ns
hsa-miR-193b	MIMAT0002819	−0.072	0.1828	0.9988	ns
hsa-miR-16	MIMAT0000069	−0.067	0.0803	0.9989	ns
hsa-miR-100	MIMAT0000098	−0.059	0.0657	0.999	ns
hsa-miR-135b	MIMAT0000758	−0.059	0.0515	0.999	ns
hsa-miR-193a	MIMAT0004614	−0.057	0.0549	0.9991	ns
hsa-miR-146b	MIMAT0002809	−0.051	0.1313	0.9992	ns
hsa-miR-125a	MIMAT0000443	−0.042	0.088	0.9994	ns
hsa-miR-181d	MIMAT0002821	−0.035	0.1467	0.9995	ns
hsa-miR-7	MIMAT0000252	−0.031	0.0815	0.9996	ns
hsa-let-7f	MIMAT0000067	−0.029	0.1111	0.9996	ns
hsa-miR-96	MIMAT0000095	−0.024	0.1182	0.9997	ns
hsa-let-7d	MIMAT0000065	−0.013	0.0577	0.9999	ns
hsa-miR-373	MIMAT0000726	−0.002	0.0811	> 0.9999	ns
hsa-miR-183	MIMAT0000261	0.0092	0.1278	0.9998	ns
hsa-miR-378	MIMAT0000732	0.0094	0.0893	0.9998	ns
hsa-let-7a	MIMAT0000062	0.0112	0.1743	0.9997	ns
hsa-miR-203	MIMAT0000264	0.0438	0.1135	0.9991	ns
hsa-let-7c	MIMAT0000064	0.0536	0.1421	0.999	ns
hsa-miR-200c	MIMAT0000617	0.0546	0.1715	0.999	ns

hsa-miR-29b	MIMAT0000100	0.0596	0.0889	0.9988	ns
hsa-miR-10a	MIMAT0000253	0.072	0.1945	0.9985	ns
hsa-miR-146a	MIMAT0000449	0.0773	0.1303	0.9983	ns
hsa-miR-199a	MIMAT0000232	0.1012	0.0518	0.9824	ns
hsa-miR-181c	MIMAT0000258	0.167	0.1058	0.4707	ns
hsa-miR-124	MIMAT0000422	0.2023	0.0932	0.1954	ns
hsa-miR-20b	MIMAT0001413	0.2176	0.0801	0.1232	ns
hsa-miR-27b	MIMAT0000419	0.2827	0.1402	0.0107	*
hsa-miR-148b	MIMAT0000759	0.3025	0.0726	0.0043	**
hsa-miR-181a	MIMAT0000256	0.3268	0.0741	0.0014	**

****, $P \leq 0.0001$; ***, $P \leq 0.001$; **, $P \leq 0.01$; *, $P \leq 0.05$; ns, $P > 0.05$

Supplementary Table S2. List of primers used.

Primer name	Sequence (5' to 3')	Type	Use
SpeI-3xFlag fwd	ATA CTA GTC CAC CAT GGA CTA CAA AGA CC	Cloning	Subcloned 3xFlag-Nurr1 into pSIN lenti expression plasmid
BamHI-Nurr1 rev	ATG GAT CCC TAG AAA GGT AAA GTG TCC A	Cloning	Subcloned 3xFlag-Nurr1 into pSIN lenti expression plasmid
EF1a fwd seq	TCA AGC CTC AGA CAG TGG TTC	Sequencing	Confirm cloning of 3xFlag-Nurr1 into pSIN plasmid
pSIN rev seq	CCC TAG ATG CAT GCG GAT CCT TCG	Sequencing	Confirm cloning of 3xFlag-Nurr1 into pSIN plasmid
pEZX-MT01 fwd	GAT CCG CGA GAT CCT GAT	Sequencing	Confirm seed region mutation
pEZX-MT01 rev	TTG GCG TTA CTA TGG GAA CAT	Sequencing	Confirm seed region mutation
CDKN1A −3969 bp RE fwd (p53-free)	ACT ATA TGC TCA GCC ATT GTG TCT GCT	ChIP qPCR	p53 ChIP of p53-free region in *CDKN1A* promoter[38]
CDKN1A −3969 bp RE rev (p53-free)	CCC TCA GCA TCA GTG TTA CCA ACC	ChIP qPCR	p53 ChIP of p53-free region in *CDKN1A* promoter[38]
CDKN1A −2242 bp RE fwd	CTG TGG CTC TGA TTG GCT TT	ChIP qPCR	p53 ChIP of *CDKN1A* promoter[38]
CDKN1A −2242 bp RE rev	CCC TTC CTC ACC TGA AAA CA	ChIP qPCR	p53 ChIP of *CDKN1A* promoter[38]
CDKN1A −11708 bp RE fwd	GAG TGG GTG GCT CAC TCT TC	ChIP qPCR	p53 ChIP of *CDKN1A* promoter[38]
CDKN1A −11708 bp RE rev	CTC GCA TCA GCA ACT CTG G	ChIP qPCR	p53 ChIP of *CDKN1A* promoter[38]
MDM2 p53 RE fwd	GAT TGG GCC GGT TCA GTG G	ChIP qPCR	p53 ChIP of *MDM2* promoter[39]
MDM2 p53 RE rev	CAC AGC TGG GAA AAT GCA TGG	ChIP qPCR	p53 ChIP of *MDM2* promoter[39]
mir-34a p53 RE fwd	ACG CTT GTG TTT CTC AGT CCG	ChIP qPCR	p53 ChIP of *mir-34a* promoter[20]
mir-34a p53 RE rev	TGG TCT AGT TCC CGC CTC CT	ChIP qPCR	p53 ChIP of *mir-34a* promoter[20]

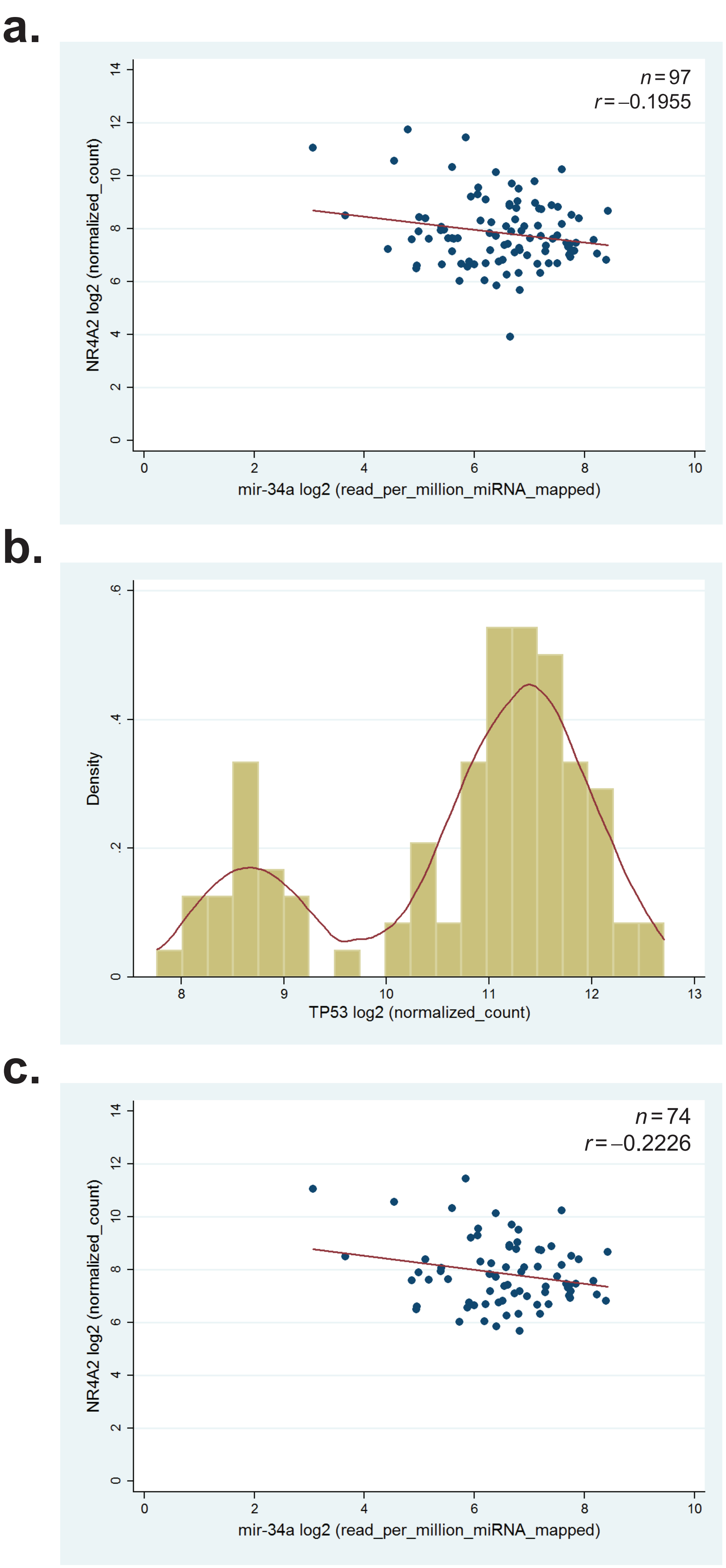

Supplementary Figure S1

a.

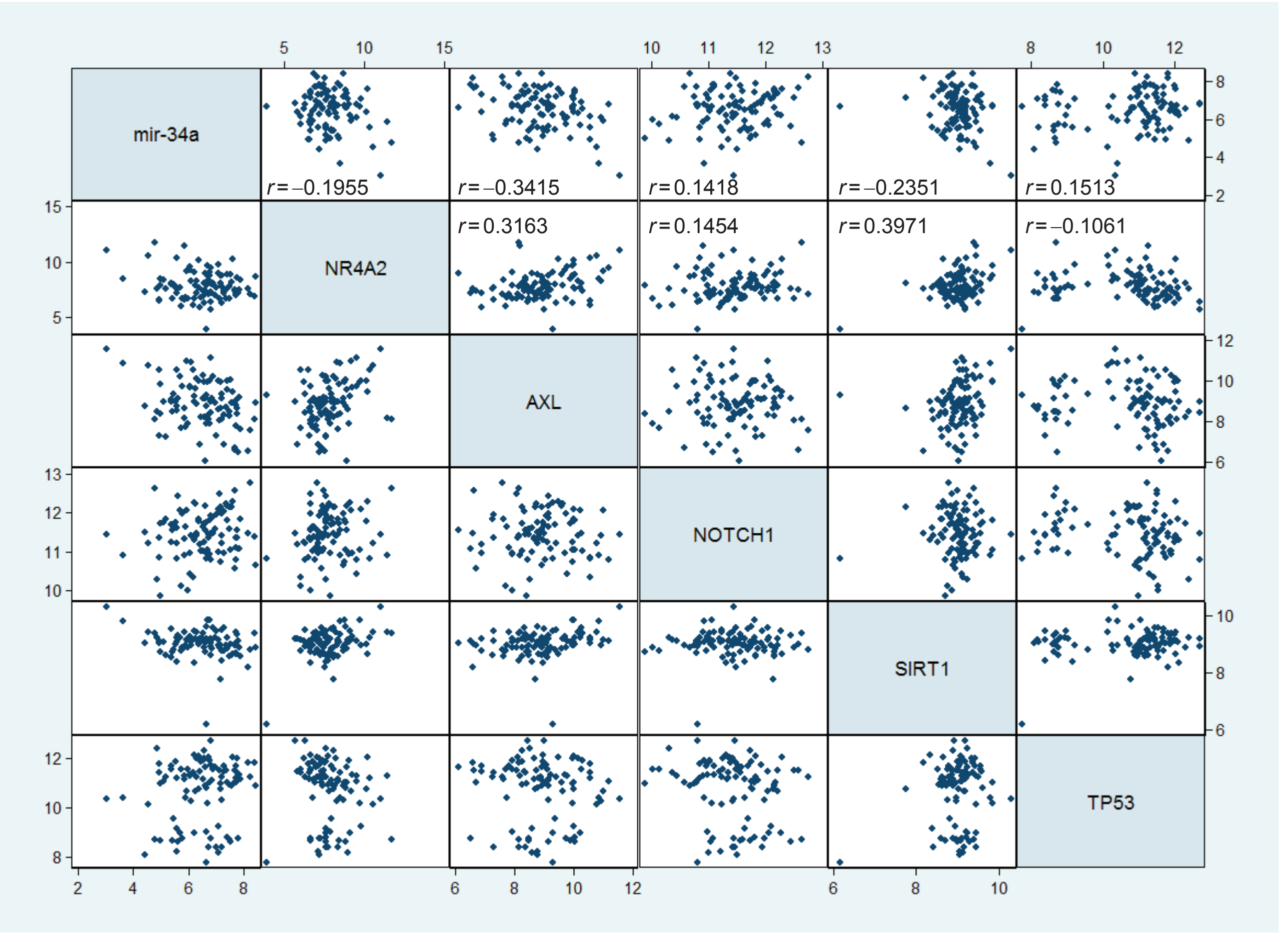

b.

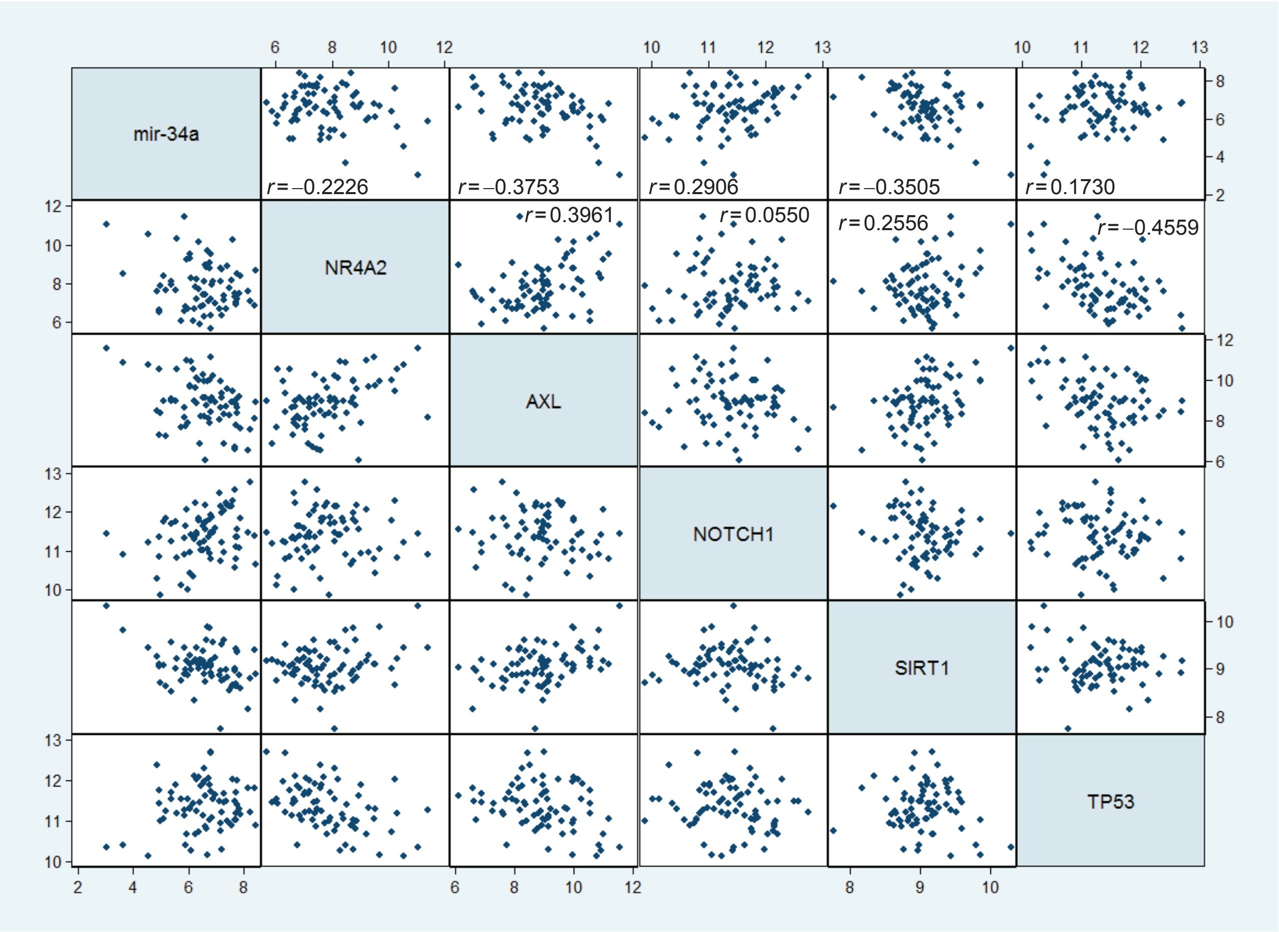

Supplementary Figure S2

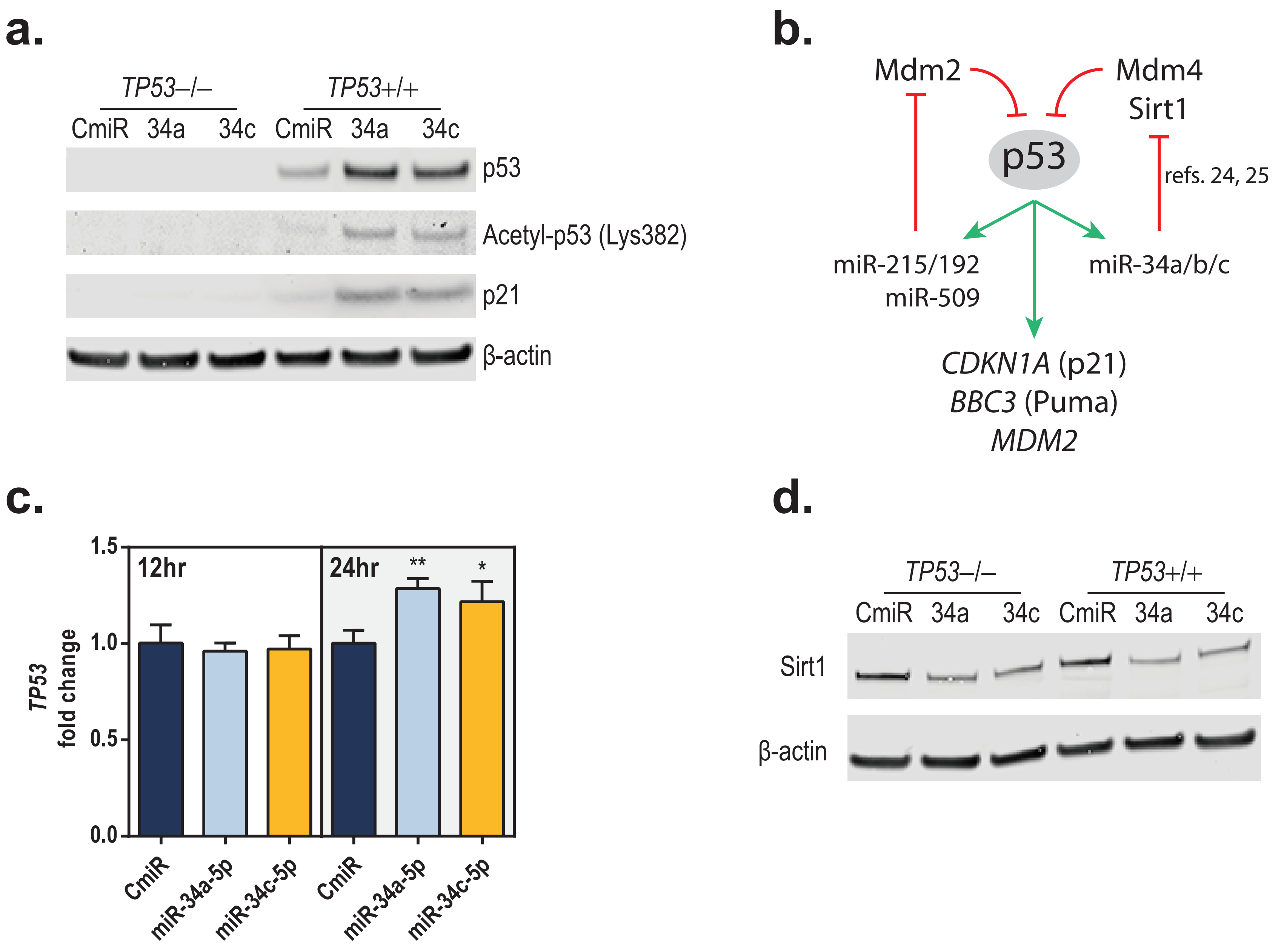

Supplementary Figure S3

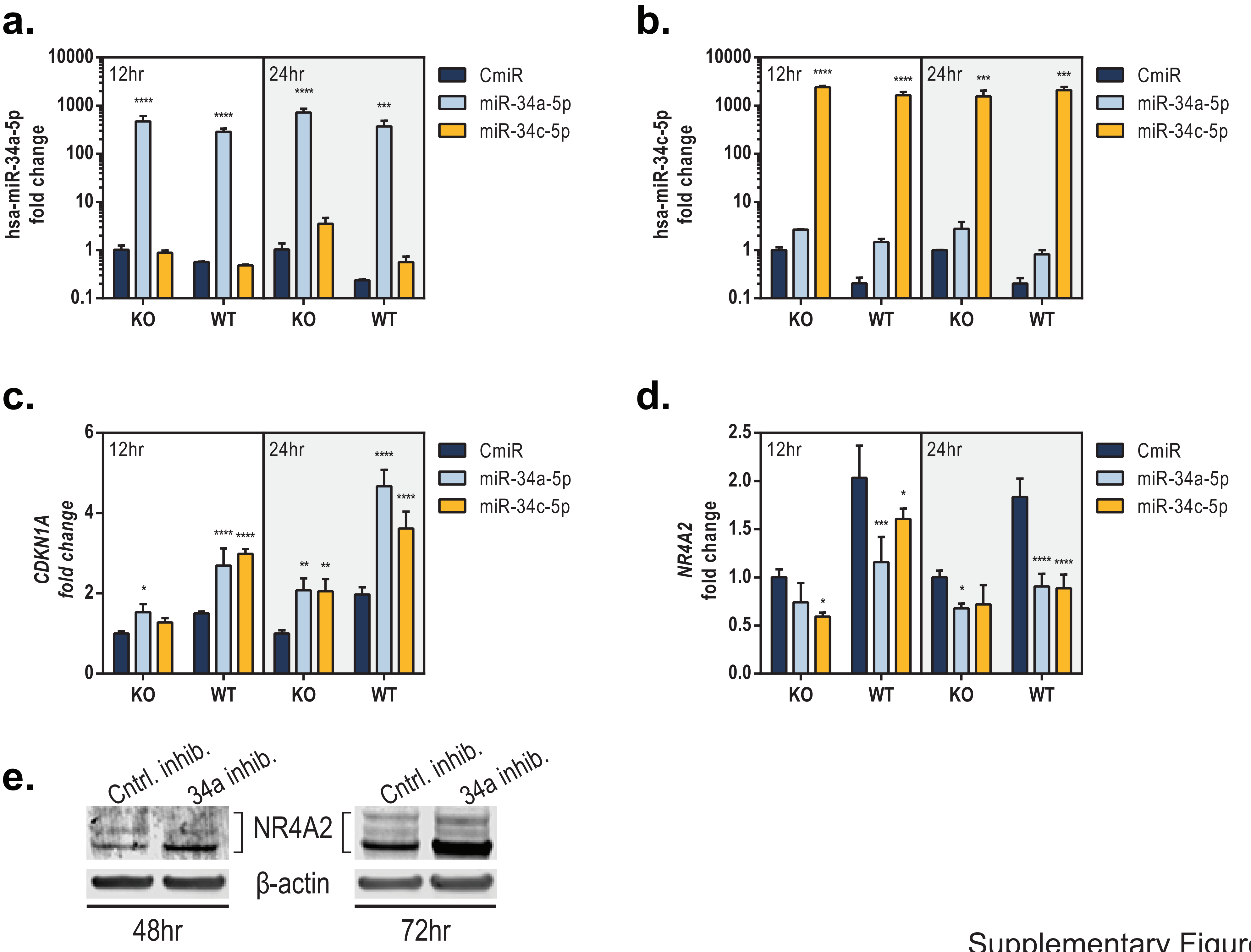

Supplementary Figure S4

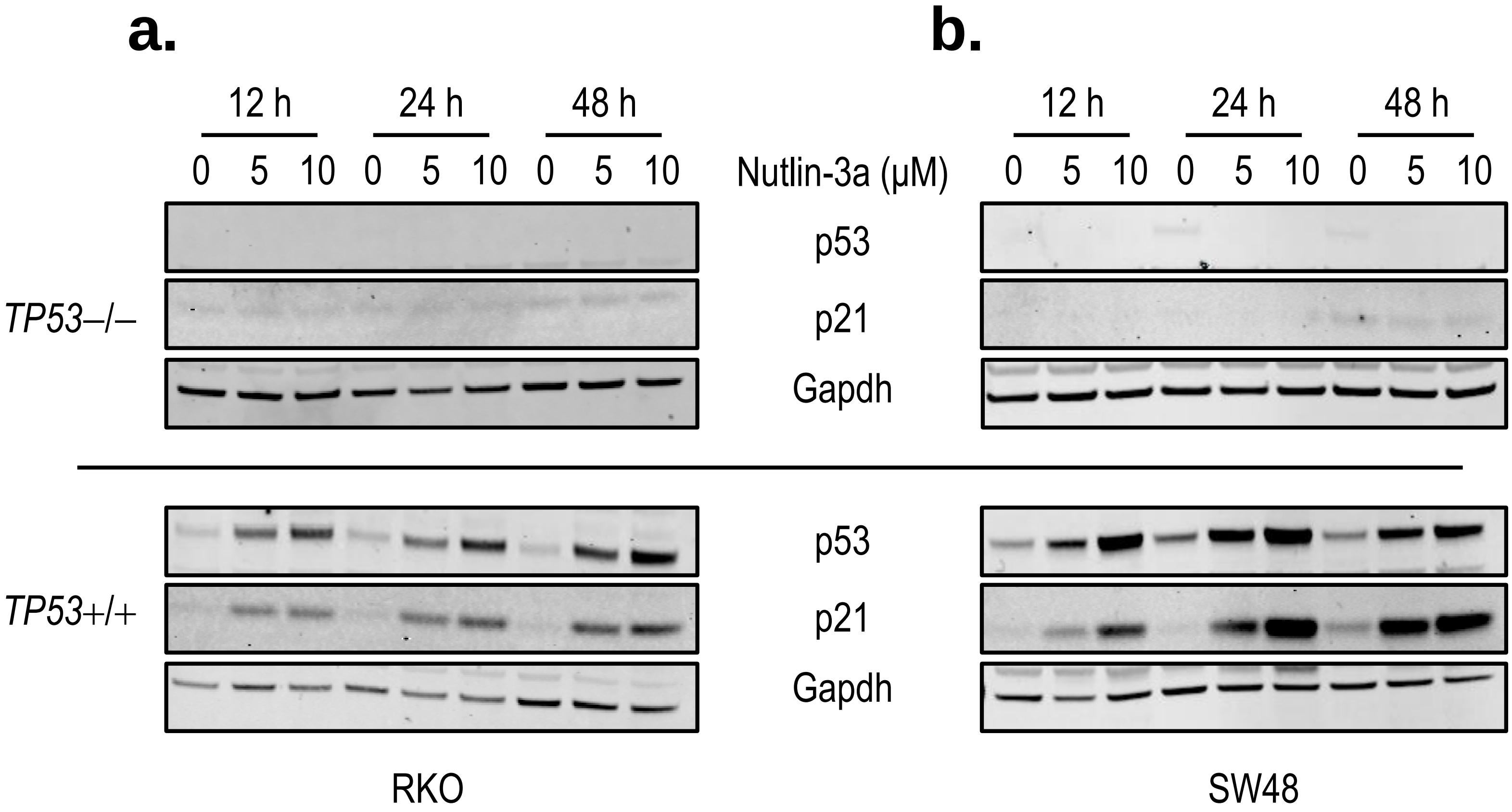

Supplementary Figure S5

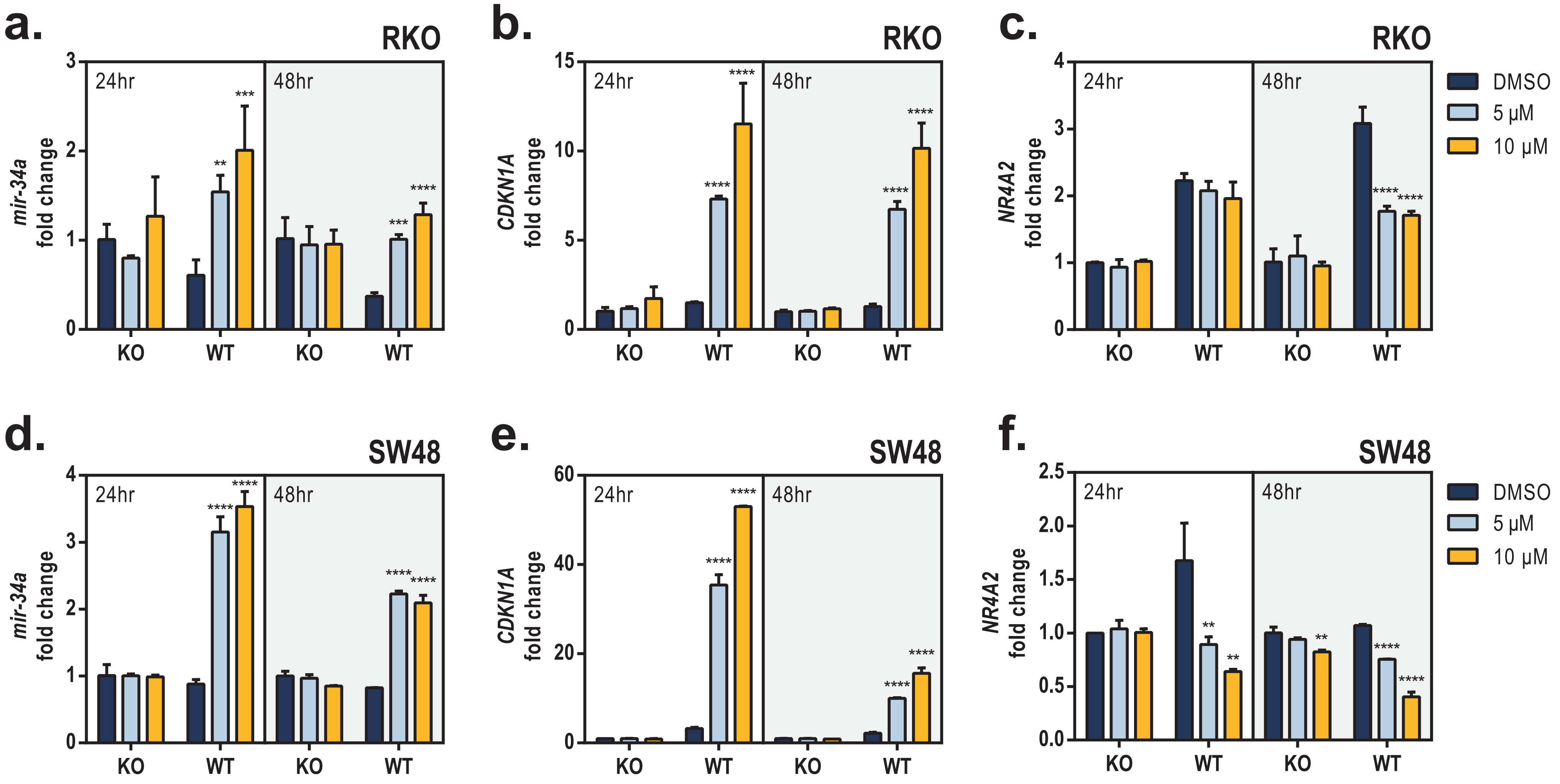

Supplementary Figure S6

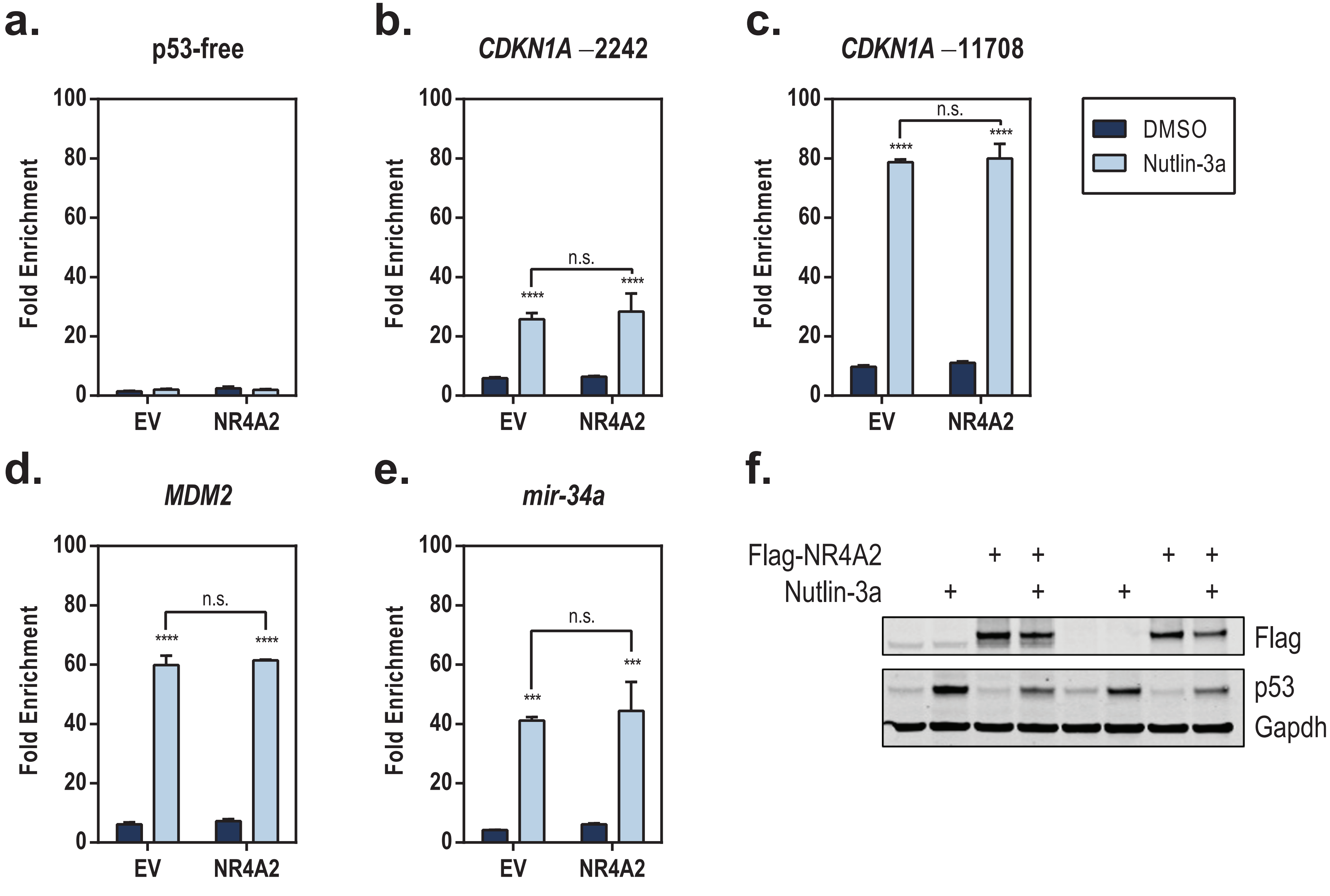

Supplementary Figure S7

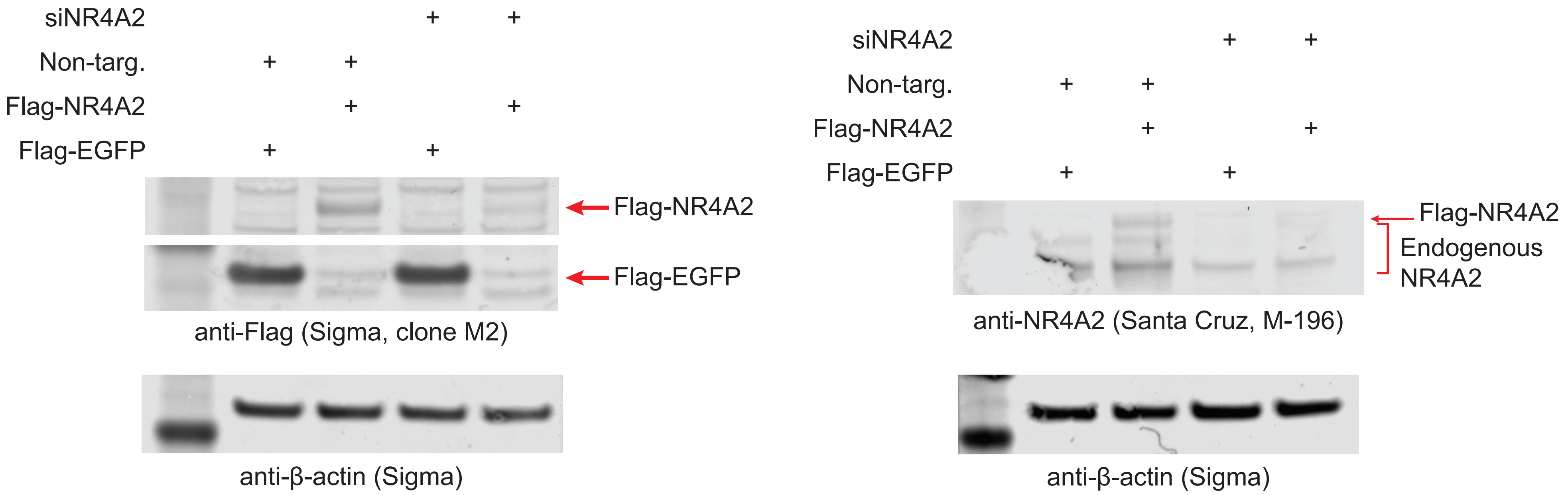

Supplementary Figure S8

Supplementary Legends

Supplementary Table S1. List of miRNAs screened. The log2 fold changes from the reporter assay screen of all the miRNAs screened are listed by miRNA ID and mirBase Accession Number. The significance of each miRNA in comparison to the transfection control (pSIF) was determined using a one-way ANOVA and Dunnett's test for multiple comparisons. ****, $P \leq 0.0001$; ***, $P \leq 0.001$; **, $P \leq 0.01$; *, $P \leq 0.05$; ns, $P > 0.05$.

Supplementary Table S2. List of primers used. The name, sequence, and use of each primer.

Supplementary Figure S1. Correlation of miR-34a and *NR4A2* expression in rectum adenocarcinoma patients. (a) Regression analysis was used to determine the correlation between expression of miR-34a and *NR4A2* in a subset of 97 patient samples from the TGCA rectum adenocarcinoma dataset. (b) *TP53* expression was determined using HiSeq data from the 97 patient samples and the distribution of expression is represented. (c) Regression analysis was used to determine the correlation between expression of mir-34a and *NR4A2* in a smaller subset of 74 patient samples with higher *TP53* expression.

Supplementary Figure S2. miR-34a correlation matrix in rectum adenocarcinoma patients. (a) Expression correlations between miR-34a and indicated genes were determined using regression analysis in a subset of 97 patient samples from the TGCA rectum adenocarcinoma dataset. (b) Regression analysis was used to determine the correlation between expression of miR-34a and indicated genes in a smaller subset of 74 patient samples with higher *TP53* expression.

Supplementary Figure S3. Overexpression of miR-34 increases p53 protein and acetylation levels. (a) Control (labeled as CmiR) or miR-34a-5p (34a) or miR-34c-5p (34c) mimics (10 nM) were transfected into HCT116 isogenic cell lines for 48 h, after which SDS-PAGE gel electrophoresis and Western blot analysis of the indicated proteins from whole cell lysates (45 µg) was performed. (b) A schematic of the previously described positive-feedback mechanisms involving p53 and its transcriptionally regulated miRNAs. (c) Expression of *TP53* was determined using a TaqMan qPCR probe after transfection of control (CmiR), miR-34a-5p, or miR-34c-5p mimics (10 nM) into HCT116 wild-type (WT) cells for the times indicated. Gene expression was normalized to *GAPDH*. The value for the CmiR-transfected WT cells at each time point was set as 1. The statistical significance of the results for miR-34 transfections, compared to those for the control, at each time point was calculated using a one-way ANOVA and Dunnett's test for multiple comparisons.**, $P \leq 0.01$; *, $P \leq 0.05$. (d) Whole-cell lysates (45 µg) from HCT116$^{TP53-/-}$ (*TP53–/–*) and HCT116 wild-type (*TP53+/+*) cells transfected for 48 h with CmiR, miR-34a-5p (34a), or miR-34c-5p (34c) mimics were assessed for expression of Sirt1 and β-actin protein by performing SDS-PAGE gel electrophoresis and Western blot analysis.

Supplementary Figure S4. Overexpression of miR-34 decreases *NR4A2* in RKO colorectal cancer cells. Mature miR-34a-5p and miR-34c-5p mimics (10 nM) were transfected into RKO$^{TP53-/-}$ (KO) or RKO wild-type (WT) cells for 12 or 24 h. Expression of hsa-miR-34a-5p (a), hsa-miR-34c-5p (b), *CDKN1A* (c), and *NR4A2* (d) was determined using TaqMan qPCR probes. Mature miRNA expression was normalized to *RNU48*, and *CDKN1A* and *NR4A2* were normalized to *GAPDH*. The value for the CmiR-transfected KO cells at each time point was set

as 1. The statistical significance of the results for miR-34 transfections for each cell line, compared to those for the control, at each time point was calculated using a two-way ANOVA and Dunnett's test for multiple comparisons. ****, $P \leq 0.0001$; ***, $P \leq 0.001$; **, $P \leq 0.01$; *, $P \leq 0.05$. (e) RKO WT cells were transfected with control or miR-34a inhibitor for indicated time points. Total protein lysates from 48hr (45 µg) or 72hr (70 µg) samples were assessed for expression of NR4A2.

Supplementary Figure S5. Nutlin-3a enhances p53 and p21 protein levels in RKO and SW48 colorectal cancer cell lines. RKO (a) and SW48 (b) colorectal cancer cell lines that are isogenic for p53 expression (*TP53*−/−, top; *TP53*+/+, bottom) were treated for the indicated time periods with vehicle (DMSO) (0 µM) or Nutlin-3a (5 or 10 µM). Whole-cell lysates were assessed for expression of p53, p21, and Gapdh protein by performing SDS-PAGE gel electrophoresis.

Supplementary Figure S6. p53 activation by Nutlin-3a decreases *NR4A2* in RKO and SW48 cell lines. RKO$^{TP53-/-}$ (KO) and RKO wild-type (WT) (a–c) or SW48$^{TP53-/-}$ (KO) and SW48 wild-type (WT) cells (d–f) were treated with vehicle control (DMSO) or Nutlin-3a (5 or 10 µM) for 24 or 48 h. Expression of *mir-34a* (a, d), *CDKN1A* (b, e), and *NR4A2* (c, f) was determined using TaqMan qPCR probes (normalized to *GAPDH*). The value for the DMSO-treated KO cells at each time point was set as 1. The statistical significance of the results obtained with Nutlin-3a treatments for each cell line, compared to those obtained with DMSO, for each time point was calculated using a two-way ANOVA and Dunnett's test for multiple comparisons. ****, $P \leq 0.0001$; ***, $P \leq 0.001$; **, $P \leq 0.01$.

Supplementary Figure S7. Overexpression of NR4A2 does not affect binding of p53 to target gene promoters. HCT116 wild-type cells were transduced with empty vector (EV) or 3xFlag-NR4A2 (NR4A2) lentivirus. After 16 h, the medium was changed and the cells were grown in culture for a total of 48 h. The cells were then reseeded into 150-mm^2 flasks and treated for 6 h with vehicle (DMSO) or Nutlin-3a (10 µM). The chromatin immunoprecipitation protocol was performed and the occupancy of p53 at the p53-free (a), *CDKN1A* −2242 (b), *CDKN1A* −11708 (c), *MDM2* (d), and *mir-34a* (e) promoter regions is shown (represented as fold-enrichment over IgG). (f) Nuclear-enriched extracts used as input for the ChIP were resolved on a 4–12% SDS-PAGE gradient gel and probed with antibodies against Flag (indicating NR4A2), p53, and Gapdh. The statistical significance of the results was determined using a two-way ANOVA with Tukey's multiple comparison test. The significance of the differences within each transduction group (****, $P \leq 0.0001$; ***, $P \leq 0.001$) and between transduction groups (n.s., $P > 0.05$) is represented.

Supplementary Figure S8. Validation of an anti-NR4A2 antibody. HCT116$^{TP53-/-}$ cells were transfected with nontargeting siRNA (Non-Targ.) or si*NR4A2* (20 nM) in 60mm^2 dishes. After 24 h of siRNA transfection, 3xFlag-EGFP (Flag-EGFP) or 3xFlag-NR4A2 (Flag-NR4A2) were transfected for an additional 48 h. Whole cell lysates (45 µg) were resolved on a 4–12% SDS-PAGE gradient gel and transferred to PVDF membrane using wet transfer (100V constant for 1 h). Membranes were probed with antibodies against Flag (detecting overexpressed NR4A2 or EGFP), NR4A2 (detecting both endogenous NR4A2 and Flag-NR4A2), and β-actin. The levels

of endogenous NR4A2 (detected using anti-NR4A2) and Flag-NR4A2 (detected using either anti-NR4A2 or anti-Flag) decreased in response to siNR4A2 but not Non-Targ.
